# Social media impact and smartwatch monitoring: Prevalence and early markers of PTSD and anxiety following mass traumatic events

**DOI:** 10.1371/journal.pmen.0000195

**Published:** 2025-09-10

**Authors:** Dan Yamin, Shahar Lev-Ari, Merav Mofaz, Ron Elias, Sharon Toker, David Spiegel, Matan Yechezkel, Margaret L. Brandeau, Erez Shmueli

**Affiliations:** 1 Department of Industrial Engineering, Tel Aviv University, Tel Aviv, Israel; 2 Department of Management Science and Engineering, Stanford University, Stanford, California, United States of America; 3 Wizermed D.H. LTD, Zoran, Israel; 4 Department of Health Promotion, Tel Aviv University, Tel Aviv, Israel; 5 Coller School of Management, Tel Aviv University, Tel Aviv, Israel; 6 Department of Psychiatry and Behavioral Sciences, Stanford University, Stanford, California, United States of America; 7 MIT Media Lab, MIT, Cambridge, Massachusetts, United States of America; IISER Bhopal: Indian Institute of Science Education and Research Bhopal, INDIA

## Abstract

We investigated PTSD, anxiety, and stress in individuals indirectly exposed to the October 7 events in Israel. Our study utilized data from a three-year prospective study of 4,806 smartwatch users who completed daily questionnaires, and a panel study surveying 2,536 individuals twice. Stress symptoms were measured daily, while PCL-5 and GAD-7 questionnaires assessed PTSD and anxiety prevalence. After October 7, stress levels soared, with PTSD prevalence reaching 22.9-36.0% at 7–8 weeks post-event, and remaining exceptionally high at 15.9-24.7% after 7 months. We found a strong correlation between increased PTSD risk and extensive media consumption. Smartwatch data revealed early PTSD markers such as pronounced increases in stress levels and declines in mood, physical activity, and sleep quality within the first week. This study underscores the significant impact of media exposure on PTSD development and the value of continuous physiological monitoring for early detection of PTSD after mass traumatic events.

## Introduction

Exposure to traumatic events can lead to the development of acute stress disorder and, subsequently, post-traumatic stress disorder (PTSD) [1]. While early detection and intervention are crucial for preventing the progression of PTSD [1], there are several gaps in the current understanding of PTSD development, particularly in the context of indirect exposure to mass traumatic events—such as the October 7 attacks, the 9/11 events in the US, or the Ukraine-Russia war—through media outlets.

First, the prevalence and persistence of PTSD among individuals indirectly exposed to traumatic events remain unclear. With the rise of social media and the increasing availability of graphic content, it is essential to understand how this type of exposure may impact mental health outcomes [2]. For example, exposure to graphic images from the Boston Marathon bombing in 2013, or the Paris terrorist attacks in 2015, were associated with post-traumatic stress symptoms [2,3]. However, as such large-scale attacks are relatively rare, it is challenging to assess their impact. Second, the relationship between the extent of media consumption, particularly graphic content, and the risk of developing PTSD needs further investigation [2]. While previous research indicates that indirect exposure to trauma can precipitate PTSD symptoms [2,4,5], the specific role of media consumption habits in this process has not been well established, as many of the media outlets we currently use were not heavily used at the time (i.e., TikTok, Instagram). Third, the preceding physiological and behavioral signs of PTSD development among individuals indirectly exposed to trauma through media have not been well characterized, as most studies relied on self-reports rather than on objective measures. Identifying these early indicators could help in developing targeted interventions and prevention strategies.

The events of October 7, 2023, which involved a series of attacks across Israeli municipalities resulting in significant casualties, sexual abuse, abductions of civilians from their homes and mass evacuation [6], provided a unique opportunity to investigate these research gaps in a natural experimental setting. The graphic coverage of the October 7 events on social media platforms like Telegram and TikTok was extremely vivid and detailed, and exceeded the official reports in the news, as terrorists used GoPro cameras to record and distribute their actions via social media platforms. As such, these documented attacks allow for an examination of the impact of indirect trauma exposure through current uncensored and unsupervised channels of media on PTSD development.

Previous research has shown that the ‘impact phase’—the initial period post-trauma—is critical in the development of PTSD, and there is evidence that autonomic arousal, as indicated by elevated resting heart rate and lower cortisol levels, can predict the development of PTSD [7]. Physiological stress responses, including the sympathetic adrenal medullary system and the hypothalamic-pituitary-adrenal axis, are designed for rapid response to stressors. When stress becomes chronic or repeated, the body engages in allostasis—its ability to dynamically adjust to changing conditions. However, prolonged activation of these adaptive mechanisms can result in adverse physiological effects, such as hypertension and sleep disruption, referred to as allostatic load, leading to a debilitating neuroendocrine pattern [8]. Neuroplasticity in brain regions involved in memory and emotion, such as the hippocampus, can strengthen pathways that trigger ongoing physiological stress responses and hyperarousal, laying the groundwork for PTSD [9]. Prior prospective studies have shown an evolution of PTSD symptoms over time [10] and have identified factors that may predict emotional distress following traumatic events [11]. The October 7 events allow us to further understand how PTSD develops and the extent to which these physiological stress responses trigger PTSD symptoms following the traumatic event.

To address these research gaps and leverage the natural experiment setting provided by the October 7 events, our study had three main aims. First, we sought to assess the prevalence and persistence of stress symptomatology, PTSD, and anxiety levels among individuals indirectly exposed to the October 7 events. Second, we aimed to identify early physiological and behavioral patterns associated with PTSD development among individuals indirectly exposed to trauma. Third, we aimed to investigate the relationship between the amount of media consumption, and the consumption of gory graphic content, and the risk of developing PTSD.

To achieve these aims, we analyzed data from two studies. In the prospective study, we analyzed data from an ongoing three-year observational prospective study of 4,806 participants who were provided with smartwatches and tasked with completing daily questionnaires using a dedicated mobile application. Complementing this, we conducted a panel study involving 2,536 participants, ensuring a representative sample of the adult Jewish population in Israel.

## Materials and methods

### Study design and participants

#### Panel study.

**Fig 1:**
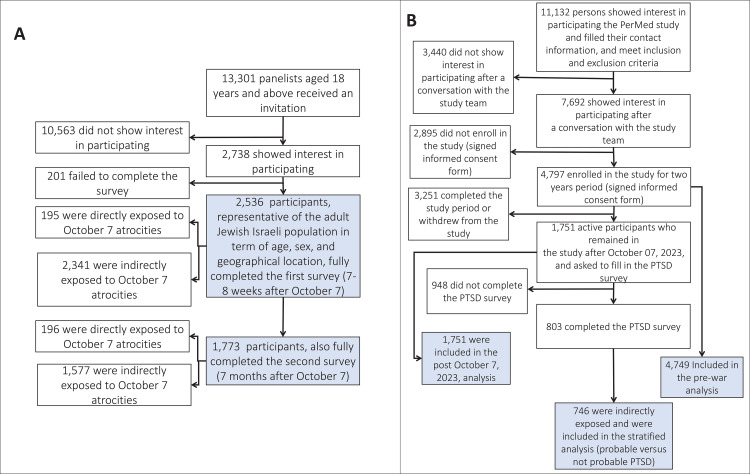
Study participants. (A) panel study, (B) prospective study.

For the panel study, we hired a professional survey company, Sarid Research Institute, to recruit 2,536 participants who completed the first survey between November 23, 2023 and December 3, 2023 (Fig 1A). All participants were invited to complete a follow-up survey between May 2, 2024 and May 21, 2024. Of the original panel of 2,536 participants, 1,773 participants completed the second survey (69.9%), and 1,577 of them (i.e., those who were not directly exposed to trauma) were included in the follow-up analysis. Participants were 18 years of age or older and representative of the adult Israeli Jewish population in terms of age, sex, and geographical location (S1 Appendix). Recruitment was conducted via email using a preregistered panelist database. The survey company ensured that all participants met the study’s eligibility criteria. To maintain sample representativeness, invitations were systematically distributed in a staggered, quota-based manner. Recruitment continued for each subgroup—defined by age, sex, and geographical location—until the corresponding quotas were fulfilled, ensuring the final sample accurately reflected the target population.

For inclusion in the panel study, participants were required to be 18 years or older, enrolled in the survey company’s database of panelists, and willing to consent to the specified terms of the study (S2 Appendix). Panelists were excluded if they failed to complete the study requirements.

#### Prospective study.

The ongoing PerMed prospective observational study included 4,806 participants aged 18 years and older who were recruited starting November 1, 2020, from all across Israel [12–16] (Fig 1B; S1 Appendix). As of November 23, 2023, a total of 1,751 participants remained active in the study. Participant recruitment was conducted via advertisements on social media and word-of-mouth. Each participant signed an informed consent form after receiving a comprehensive explanation of the study from the professional survey company, Sarid Research Institute. Participants were invited to complete an online PTSD survey 7–8 weeks after October 7. Of the original sample of 4,806 participants, 803 completed the online PTSD survey; among these, 746 were not directly exposed to trauma.

To be included in the PerMed study, participants needed to be 18 years or older, using their own smartphone, and able to give written informed consent by themselves. Participants were excluded if they knew that they would be outside of Israel for more than three months continuously at any point for the two years after enrollment. Participants were also excluded if they were students or employees of the principal investigator.

### Procedures

#### Panel study.

Upon enrollment, roughly eight weeks after October 7 events, participants were provided with written information about the nature of the study. Participants were asked to complete online PTSD and anxiety surveys, to better understand the effects of the October 7 events and to detect cases of PTSD and anxiety at the nationwide level. At this stage, participants were also requested to provide demographic information, including age, sex, household size, educational background, monthly income, religious level, media consumption behavior such as the number of hours they watched news and the number of graphic videos they watched, and PTSD background (S1 Appendix). Seven months after October 7, participants were asked to complete a second follow-up online survey on PTSD and anxiety. This survey aimed to better understand the long-term prevalence of estimated PTSD and anxiety levels in the Israeli population.

The online surveys incorporated the Post-traumatic Stress Disorder Checklist (PCL-5), a self-report questionnaire designed to assess the 20 symptoms outlined in the DSM-5 criteria for PTSD. The PCL-5 serves various purposes, including screening individuals for PTSD and making provisional diagnoses [17]. It is widely used for clinical and research purposes; psychometrically, the PCL-5 demonstrates strong internal consistency (α = .94), high test-retest reliability (r = 0.82), and strong convergent validity (r = 0.74 to 0.85) [17]. The nine or more required symptoms of Acute Stress Disorder (ASD) are basically the same as those of PTSD in the DSM-5TR, are not constrained to being distributed among separate categories of intrusion, negative mood, dissociation, avoidance and arousal, and must occur three days to one month after trauma exposure. ASD often precedes but is not the only pathway to developing PTSD.

The surveys also utilized the General Anxiety Disorder (GAD) 7-item questionnaire (GAD-7) to identify cases of GAD and measure anxiety symptom severity. The tool is also widely used as a screening measure of panic, social anxiety, and PTSD. The questionnaire is considered valid, sensitive, and specific for the diagnosis of GAD in the general population [18]. We used the Israeli Ministry of Health’s Hebrew translation of these two questionnaires.

#### Prospective study.

After enrollment in the prospective study, participants used the PerMed mobile application to complete a daily questionnaire [14,16,19]. The questionnaire allowed participants to report on stress levels (reported on a scale of 1–5, where 1 represents ‘very low,’ and 5 signifies ‘very high.’), mood (reported on a scale of 1–5 where 1 represents ‘awful’ and 5 signifies ‘excellent’), and sleep quality (reported on a scale of 1–5 where 1 represents ‘awful’ and 5 signifies ‘excellent’) (S1 Appendix). Participants who were active in the study after the October 7 events were asked to fill in an online PTSD survey eight weeks later (S2 Appendix).

Participants in the prospective cohort were equipped with Garmin Vivosmart 4 fitness trackers. Among other features, the smartwatch collects data on all-day heart rate, heart rate variability (HRV)-based stress [20], step counts, sleep duration, and sleep-level classification, including light, deep, rapid eye movement (REM), and awake periods during sleep [12] (S1 Appendix). We focused on these measures because they provide continuous information on the well-being of an individual. Additionally, individuals diagnosed with PTSD may encounter nightmares or insomnia and often report a reduction in sleep time due to waking up earlier than desired, restlessness during the night, and difficulties falling asleep, all of which impact daily life [21]. Step counts are strongly correlated with the physical activity level of an individual. Given that PTSD is often co-morbid with, and shares overlapping clinical features of, anxiety and depression, individuals diagnosed with PTSD tend to be less physically active [22].

We developed a dedicated data collection platform that collects for each participant data from the smartphone sensors and daily questionnaires via the PerMed application and the smartwatch sensors via the Garmin Health Application Programming Interface (S1 Appendix). These collected data are securely stored in Tel Aviv University facilities.

Participants received Garmin Vivosmart 4 smartwatches, and installed two applications on their mobile phones: (i) the PerMed application [14,16,19], which collects daily self-reported questionnaires; and (ii) an application that passively records smartwatch data. Participants were asked to wear their smartwatches as much as possible. Using a dedicated dashboard to monitor compliance, a survey company ensured that participants’ questionnaires were filled out at least twice a week, that their smartwatches were charged and properly worn, and that any technical problems with the mobile applications or smartwatch were resolved (S1 Appendix).

We implemented several preventive measures to minimize participant attrition and discomfort and thereby improve the quality, continuity, and reliability of the collected data. First, each day, participants who did not fill out their daily questionnaire by 19:00h received a reminder notification through the PerMed application. Second, we developed a dedicated dashboard that allowed the survey company to identify participants who repeatedly neglected to complete the daily questionnaire or did not wear their smartwatch for extended periods of time. These participants were contacted by the survey company (either by text message or phone call) and were encouraged to better adhere to the study protocol. Third, to strengthen participants’ engagement, a weekly summary report was generated for each participant, which was available inside the PerMed application. Similarly, a monthly newsletter with recent findings from published studies and useful tips regarding the smartwatch’s capabilities was sent to the participants. At the end of two years, participants received all the obtained personal insights and could keep the smartwatch as a gift (S1 Appendix).

### Outcomes

For both the prospective and panel study cohorts, we analyzed the online PTSD survey (Table S2.1 in S2 Appendix). We defined the main outcomes as PTSD and anxiety levels for each participant.

For the prospective study, we analyzed step counts, sleep duration, and awake episodes during sleep from the smartwatches. In analyzing the daily questionnaires, we calculated the daily mean of reported stress, reported mood, reported sleep quality, and reported sleep duration before and after the events of October 7. For each measure, the prespecified outcomes are the daily mean value of each measurement. We calculated the daily mean value before and after the events of October 7.

We also analyzed heart rate and HRV-based stress from smartwatches. For these measures, we calculated the hourly mean value before and after the events of October 7.

### Statistical analysis

PTSD diagnosis requires an interview conducted by a trained clinician. In this study, the outcome was derived from a screening tool. We defined PTSD as a total score of 33 on the PCL-5 questionnaire and meeting the DSM-5 diagnostic rule which requires at least: 1 B item (questions 1–5), 1 C item (questions 6–7), 2 D items (questions 8–14), 2 E items (questions 15–20). This definition aligns with the most conservative criteria, consistent with the guidelines established by the International Society for Traumatic Stress Studies [23] (S2 Appendix). In a secondary analysis, we considered a less conservative definition of PTSD, whereby it is identified by either a total score of 31 on the PCL-5 questionnaire or by meeting the DSM-5 diagnostic rule.

In line with the GAD-7 criteria, the anxiety level of participants was determined by the total score: no anxiety (0–4), mild (5–9), moderate (10–14) and severe (≥15) [18].

Only fully completed questionnaires were included in the analyses; hence, no imputation or other handling of missing data was required for either the descriptive statistics or the inferential statistics.

### Panel study

#### Descriptive statistics.

We analyzed the data of participants who had indirect exposure to the events of October 7. Specifically, differently from the DSM-5 definition of indirect exposure, we defined indirect exposure for all participants who, along with their immediate family, were not injured, killed, or abducted (S2 Appendix). To ensure specificity in attributing PTSD sources, we also excluded individuals who were forced to evacuate their homes following the October 7 events, as this stressful experience which inflicted about 200,000 citizens may also contribute to PTSD symptomatology. We evaluated the estimated prevalence of PTSD as the proportion of individuals with PTSD (as assessed in the first PTSD survey). We also stratified the PTSD prevalence estimate by (a) the duration of news information consumption during the first week after October 7 and (b) the extent of exposure to gory videos (such as videos documenting the killings carried out on October 7, or the abductions of the captives (S2 Appendix). We also evaluated the rates of exposure to gory videos on different media platforms (Instagram, TikTok, etc.). Specifically, we divided the number of participants who were exposed to gory videos on each platform by the number of participants who consumed news information using that platform. Additionally, we examined the rates of each anxiety level category. The 95% confidence intervals (CIs) were obtained under the assumption of binomial distributions.

#### Inferential statistics.

To examine the associations between PTSD/anxiety (as assessed in the first PTSD survey), duration of news consumption during the first week following October 7, and the extent of exposure to gory videos, while controlling for other explanatory variables (age, sex, educational background, religious level, socioeconomic level, and PTSD/anxiety background), we applied a logistic regression model. For both models, we derived the odds ratios and their corresponding 95% confidence intervals from the values of the regression coefficients. More details are available in S3 Appendix.

### Prospective study

*Descriptive statistics:* From the commencement of the prospective study on January 1, 2021, to April 30, 2024, we collected 2,106,435 days of smartwatch data and 787,998 self-reported questionnaires. Additionally, 803 participants filled out the PTSD online survey 7–8 weeks after October 7.

Before analyzing the data, we conducted a preprocessing step. If participants filled out the daily questionnaire more than once on the same day, only the latest questionnaire for that day was considered. The rationale behind this decision was that once a questionnaire was filled out, it was sent to the server, and could not be updated. Therefore, in the case of a filling error, participants were instructed to re-fill the questionnaire.

For a more comprehensive understanding of individual perception, we examined the reported stress levels throughout the PerMed study period. We calculated the daily moving average and the associated 95% confidence interval with a 7-day window from January 1, 2021 to April 30, 2024 for all active participants. For participants who filled out the PTSD online survey, we implemented the same procedure, stratifying them based on whether they exhibited PTSD.

We noted several significant national events that might have influenced reported stress levels, including the major Jewish holidays (Passover and Rosh Hashanah), two armed conflicts in 2022 and 2023 involving the firing of at least 1,000 rockets at the civilian population, a major political demonstration related to judicial reform that was escalated on March 26, 2023, following the dismissal of the Minister of Defense and the strike in both the public and private sectors, and the SARS-CoV-2 Omicron wave, which persisted from December 20, 2021 to May 1, 2021, with approximately 30% of the Israeli population reportedly infected and a forced lockdown [24].

For each participant who remained active after October 7, 2023, we assessed 10 well-being indicators in the week prior to October 7, 2023 (baseline period) and the week after October 7, 2023: reported mood level, reported stress level, reported sleep quality, reported sleep time, step count, distance travelled, and duration of awake and light/REM/deep sleep periods during sleep. We computed a single weighted average value for each of the two periods.

The weighted average for each participant was determined by first averaging the corresponding indicator values separately for workdays and free days (weekends and national holidays). Subsequently, the weighted average of these two values was calculated, assigning a weight of 5/7 to workdays and 2/7 to free days. The rationale for computing this weighted average is further explained elsewhere [12]. In essence, we identified a weekly rhythm across various indicators, with free days exhibiting different mean daily values than workdays. Given the relatively short duration of the examined time periods and variations in the number of free days (e.g., holidays), we aimed to correct for potential biases.

Finally, for each of the 10 indicators, we calculated the average over all participants and the associated 95% confidence interval for each period, as well as the average difference.

For participants who filled out the PTSD online survey and were indirectly exposed, we repeated the above analyses, stratifying participants based on whether they exhibited PTSD.

*Inferential statistics:* To investigate the statistical significance of differences between the two periods and between the exhibition of PTSD and no exhibition, we used a mixed ANOVA design. For each of the above-noted 10 indicators, we used a separate mixed ANOVA test. In each test, the considered indicator served as the dependent variable. For the independent variables (main factors), the within-subjects factor was the period (the week prior to October 7, 2023 and the week after October 7, 2023(. The between-subjects factor was the exhibition of PTSD (as assessed in the first PTSD survey). To reduce the risk of inflated Type I errors due to multiple comparisons, we applied the Benjamini-Hochberg procedure. More details are available in S3 Appendix. All statistical analyses were performed using Python 3.8 with the following libraries: statsmodels 0.14.1, scipy 1.8.0.

### Ethics

The panel study and the prospective study protocols were approved by the Tel Aviv University Institutional Review Board (0007545–1). Before participating in the panel study, all subjects were advised, in writing, as to the nature of the study. All data were de-identified and no personally identifiable information was gathered.

## Results

### Levels of stress, anxiety, and PTSD

The first aim of our study was to assess the degree to which indirect exposure to an acute and severe large-scale stressor can trigger the occurrence of PTSD, anxiety, and stress symptoms. On the morning of October 7, 2023, during a time usually marked by holiday calm and weekend relaxation, a series of unexpected atrocities jolted the Israeli population, resulting in the first 48 hours in about 1,450 dead and 11,500 wounded civilians and soldiers, and 250 people being taken hostage. In addition, 200,000 civilians were evacuated from their homes. Regardless of political views, this event serves as a natural experiment, triggering unprecedented levels of stress, anxiety, and post-traumatic stress disorder responses. The extent of this upheaval was captured through detailed data from daily self-reported questionnaires and a variety of physiological metrics measured by smartwatches worn by participants in our prospective study, as well as in reports by participants in the panel study.

### Acute stress symptomatology in the prospective study

**Fig 2:**
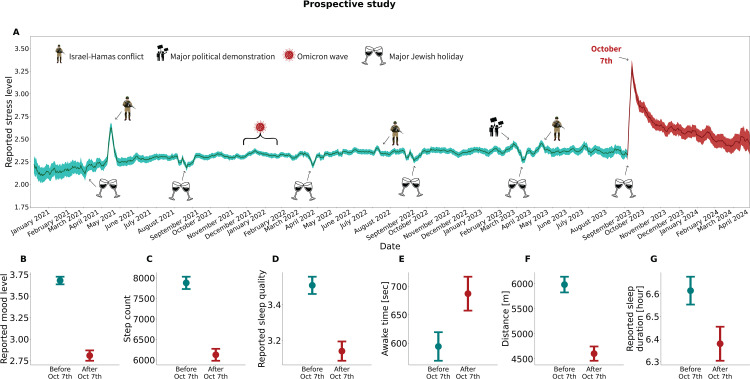
Prospective study measurements before and after October 7. (A) Mean daily reported stress levels for the prospective study’s active participants for the period January 1, 2021, to April 30, 2024. The stress levels were reported by participants as part of 787,998 daily questionnaires filled out. Stress was reported on a scale of 1 (Low) - 5 (High). The presented values are the daily moving average and the associated 95% confidence interval using a 7-day window. Panels (B-G) compare the measures recorded in a one-week period before October 7 (September 30, 2023 to October 6, 2023) with those from the week after. Mean values and 95% confidence intervals are presented. In all panels, the color red represents data recorded after October 7, and green represents data recorded before October 7.

Data based on 787,998 daily questionnaires filled out by 4,806 participants between January 1, 2021 and April 30, 2024 revealed that reported stress levels after October 7 were unprecedented (a maximum level of 3.45 [95% CI: 3.3-3.6], on a scale of 1–5; ‘very low’– ‘very high’) and far exceeded those observed during previous events, including acute stressors that occurred during this period (Fig 2A). This includes the deadliest and most contagious COVID-19 wave, during which 30% of the Israeli population tested positive over a short period of three months (a maximum stress level of 2.44 [95% CI: 2.4-2.48]); widespread political protests sparked by a controversial judicial reform, culminating in the dismissal of an Israeli minister and strikes across private and public sectors (a maximum level of 2.6 [95% CI: 2.54-2.66]); and intense military engagements, with over 1,000 rockets launched in 2022 and 2023 from Gaza towards multiple Israeli cities (a maximum level of 2.94 [95% CI: 2.83-3.05]).

To explore the adverse effect of October 7 on objective and subjective stress responses, we compared the levels of 10 well-being indicators in the week that preceded October 7 (September 30 to October 6) and the week that followed October 7 (October 7 to October 14) (Table D in S4 Appendix). Fig 2B–G, depicts the six indicators (in addition to reported stress level) for which the difference between the two periods was found to be statistically significant (Table F in S4 Appendix). As shown in Fig 2B–G, there was a marked decline in reported mood level (3.68 [95% CI: 3.59-3.77] vs. 2.81 [95% CI: 2.69-2.93], on a scale of 1–5); decreased physical activity, as evidenced by a significant drop in daily step count (7,881 [95% CI: 7,588-8,175] vs. 6,127 [95% CI: 5,841–6,411] steps) and distance travelled (5,983 [95% CI: 5,676-6,290] vs. 4,605 [95% CI: 4,326-4,885] meters); and a distinct decrease in sleep quality, as evidenced by a decrease in reported sleep quality (3.51 [95% CI: 3.41-3.60] vs. 3.14 [95% CI: 3.03-3.25], on a scale of 1–5), a decrease in reported sleep time (6.61 [95% CI: 6.49-6.74] vs. 6.38 [95% CI: 6.23-6.53] hours), and an increase in awake time during night sleep (594.25 [95% CI: 544.84-643.66] vs. 687.35 [95% CI: 628.82-745.88] seconds).

Further evidence for intense physiological arousal during the first 72 hours after October 7 can be found in Figs A and B in S4 Appendix, where compared to the baseline level measured on the same days and hours one week before October 7, there was a significant increase in heart rate, as well as in HRV-based stress. These changes were comparable to the reactions we previously observed following COVID-19 vaccinations [13].

### Levels of PTSD and anxiety in the panel study

Next, we evaluated the extent to which the atrocities had post-traumatic effects on Israeli civilians who were indirectly exposed to the atrocities. To do so, we conducted a survey among a panel of 2,536 participants 7–8 weeks after October 7. Participants were representative of the adult Jewish population in Israel by age, sex, and geographical location (Table A in S4 Appendix). We measured PTSD using the Post-traumatic Stress Disorder Checklist (PCL-5). The PCL-5 questionnaire [17] has previously demonstrated strong internal consistency, high test-retest reliability, and strong convergent validity with the gold standard PTSD diagnosis. Among the 2,341 participants who were indirectly exposed to the atrocities and filled out the first PTSD survey, the estimated prevalence of PTSD was 22.90% [95% CI: 21.19%-24.60%] using the most conservative case definition (Table 1).

**Table 1:** Estimated PTSD prevalence and anxiety levels among participants in the panel study cohort who were indirectly exposed to October 7 atrocities.

		First survey	Second survey
**Total N**		2,341	1,388
**Estimated PTSD prevalence**^**†**^ % (95% CI^*^)		22.90% (21.19%-24.60%)	15.98% (14.20%-17.82%)
**Estimated anxiety levels**^§^ % (95% CI^*^)	**No**	42.16% (40.15%-44.17%)	58.47% (56.06%-60.88%)
	**Mild**	30.63% (28.75%-32.51%)	25.17% (23.02%-27.33%)
	**Moderate**	17.77% (16.23%-19.35%)	11.35% (9.83%-12.94%)
	**Severe**	9.44% (8.29%-10.64%)	5.01% (3.93%-6.09%)
	**Any (mild-to-severe)**	57.84% (55.83%-59.85%)	41.53% (39.12%-43.94%)
	**Moderate-to-severe**	27.21% (25.42%-29.00%)	16.36% (14.58%-18.20%)

* 95% CI was obtained under the assumption of binomial distribution.

^†^Estimated prevalence based on the most conservative criteria defined as a total score of 33 on the PCL-5 questionnaire and meeting the DSM-5 diagnostic rule which requires at least: 1 B item (questions 1–5), 1 C item (questions 6–7), 2 D items (questions 8–14), 2 E items (questions 15–20).

^§^In line with the GAD-7 criteria, the anxiety level of participants was determined by the total score: no anxiety (0–4), mild (5–9), moderate (10–14), and severe (≥15).

Seven months after October 7 (during May 2024), all participants were invited to complete a follow-up survey. Of the initial sample, 1,773 completed the second PTSD survey; 1,577 of these participants were indirectly exposed. PTSD prevalence continued to remain considerably high, with estimated PTSD prevalence of 15.98% [95% CI: 14.20%-17.82%] using the most conservative case definition. This prevalence was substantially higher than the 7.5% found 6–8 weeks after the 9/11 terror attack among New York City residents [5,25].

Estimated PTSD prevalences for the least conservative case definition are presented in S4 Appendix.

We also measured anxiety symptoms using the General Anxiety Disorder (GAD) 7-item questionnaire. Among those not directly exposed, the estimated prevalence of anxiety in the first online survey was 57.84% [95% CI: 55.83%-59.85%], with 27.21% [95% CI: 25.42%-29.00%] having moderate to severe anxiety (Table 1). Seven months after October 7, the estimated prevalence of anxiety remained relatively high, with 41.53% [95% CI: 39.12%-43.94%] exhibiting symptoms of anxiety.

### Physiological, psychological, and behavioral patterns associated with PTSD in the prospective study

The second aim of our study was to identify physiological, psychological, and behavioral patterns associated with the likelihood of developing PTSD following indirect exposure to a severe acute stressor. To answer this question, we asked the prospective study participants to complete the online PTSD survey (using the same PCL-5 measure) 7–8 weeks after October 7. In this sample of 746 participants who completed the survey and were indirectly exposed, 16.22% [95% CI 13.67%-18.90%] were diagnosed with PTSD assuming the most conservative criteria. Estimated PTSD prevalence for the least conservative case definition as well as estimated prevalence of anxiety levels are presented in S4 Appendix.

We found statistically significant differences in the baseline period between individuals who later exhibited PTSD and those who did not, in 7 out of the 10 considered well-being indicators (Tables E and F in S4 Appendix). Specifically, individuals who later exhibited PTSD had lower reported mood levels (3.55 [95% CI: 3.50-3.61] vs. 3.71 [95% CI: 3.66-3.76], on a scale of 1–5); higher reported stress levels (2.67 [95% CI: 2.60-2.73] vs. 2.29 [95% CI: 2.23-2.34], on a scale of 1–5); decreased physical activity, as evidenced by daily step count (7,492 [95% CI: 7,320–7,663] vs. 8,022 [95% CI: 7,844–8,200] steps) and distance travelled (5,407 [95% CI: 5,248-5,565] vs. 6,259 [95% CI: 6,057-6,461] meters); and poorer sleep, as evidenced by reported sleep quality (3.37 [95% CI: 3.31-3.43] vs. 3.58 [95% CI: 3.53-3.63], on a scale of 1–5), reported sleep time (6.35 [95% CI: 6.27-6.43] vs. 6.66 [95% CI: 6.59-6.72] hours), and light sleep time (16,797 [95% CI: 16,646–16,948] vs. 15,911 [95% CI: 15,760–16,062] seconds).

**Fig 3:**
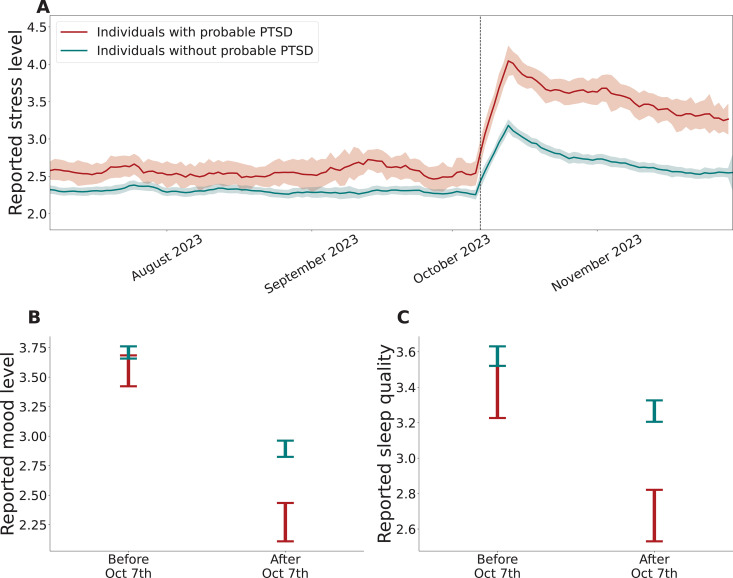
Prospective study measurements before and after October 7, stratified by PTSD status. (A) Mean daily reported stress level for the prospective study’s active participants for the period July 1, 2023 to April 30, 2024, stratified by PTSD status (as determined later using the PCL-5 questionnaire). Stress levels were reported by participants as part of the daily questionnaires on a scale of 1 (Low) - 5 (High). The presented values are the daily moving average and the associated 95% confidence interval using a 7-day window. Panels (B-C) compare the extent of change from a one-week period before October 7 (September 30, 2023 to October 6, 2023) and the week after, between individuals who later exhibited PTSD and those who did not. Mean values and 95% confidence intervals are presented.

We also found that the extent of change between the two periods (the week that preceded October 7 and the week that followed October 7) was significantly different between the two groups (individuals who will later exhibit PTSD and those who will not), in 3 out of the 10 considered well-being indicators (Tables E and F in S4 Appendix, Fig 3). Specifically, individuals who later exhibited PTSD presented a sharper increase in reported stress levels (1.31 vs. 0.85, on a 1–5 scale), and a sharper decline in reported mood level (1.28 vs. 0.82, on a 1–5 scale) and reported sleep quality (0.70 vs. 0.31, on a 1–5 scale).

### Association between exposure to news and gory videos and the prevalence of PTSD in the panel study

**Fig 4:**
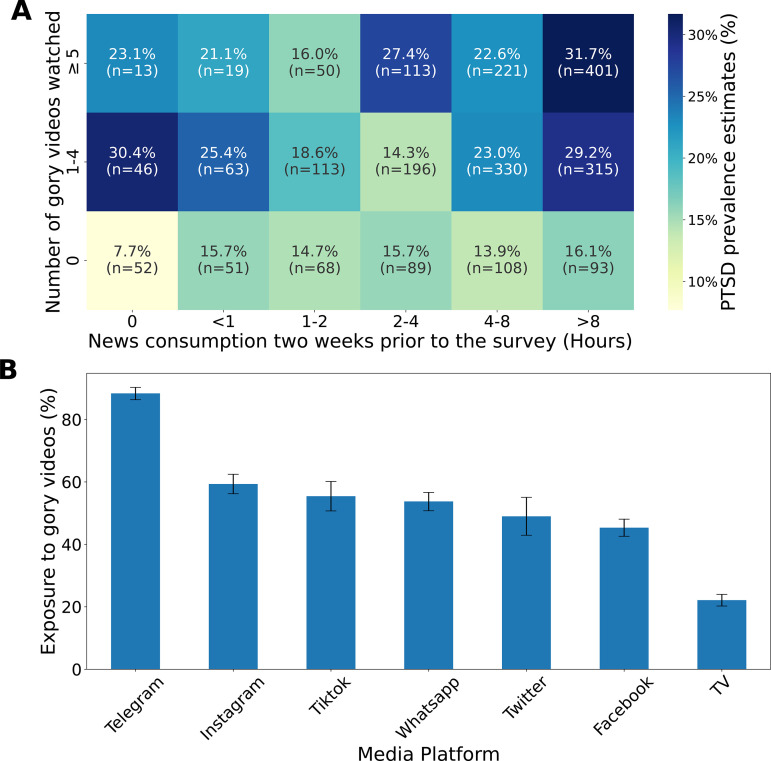
(A) Estimated prevalence of PTSD as a function of the number of hours spent watching news each day and the number of gory videos watched, in the first week after October 7. Adjusting for age, sex, educational background, religious level, socioeconomic status, and PTSD background, a logistic regression confirms that ‘news consumption’ and ‘number of gory videos watched’ are significantly associated with PTSD (p-value 0.012 and 0.003, respectively) (Table C in S4 Appendix). (B) Exposure rate to gory videos by media platforms. For each media platform, we calculated the exposure rate as the number of participants who reported watching gory videos in that media platform divided by the total number of individuals who reported using that media platform.

The third aim of our study was to assess the degree to which indirect exposure to trauma via media consumption is associated with PTSD and anxiety symptoms. In contrast to the 9/11 terror attack, where smartphones and social media platforms were not available, the October 7 events were extensively documented in real-time through GoPro cameras and disseminated on various media platforms. As such, even those who were not directly exposed to these atrocities could hear or see them. We thus investigated the relationship between indirect exposure to trauma, as represented by self-reported frequency of news consumption and number of gory videos viewed, and PTSD among panel participants (Fig 4). After adjusting for age, sex, socioeconomic status, and level of religiosity, the duration of news consumption during the first week following October 7 was found to be significantly associated with PTSD, with each additional unit of duration corresponding to a 10.4% increase in the odds of PTSD (OR = 1.104 [95% CI: 1.022–1.193], p = 0.012). Similarly, the extent of exposure to gory videos was found to be significantly associated with PTSD, with each additional unit of exposure corresponding to a 25.9% increase in the odds of PTSD (OR = 1.259 [95% CI: 1.080–1.468], p = 0.003) (Table B in S4 Appendix). For instance, estimated PTSD prevalence among those who did not watch any gory videos or news was 7.7%, compared to 31.7% among those who watched more than eight hours of news per day and viewed five or more gory videos during the first week after the event (Fig 4A). Among the various media platforms, Telegram presented the highest exposure rate to gory videos at 88.4%, followed by Instagram and TikTok at 59.3% and 55.4%, respectively (Fig 4B). A similar analysis examining the association between the duration of news consumption and the extent of exposure to gory videos to anxiety, after adjusting for age, sex, socioeconomic status, and level of religiosity is available in Table C in S4 Appendix.

## Discussion

In today’s reality, where detailed, unfiltered, graphic, and prolonged media coverage of massive traumatic events is common, we find that PTSD rates among those indirectly exposed can be substantially high, reaching 23–36%, and remain high even seven months later, at 14–22%. These rates are exceptionally high, compared to those reported by health professionals who encounter trauma in their professional duties [26–28]. Specifically, the hours spent watching news, and the number of gory videos viewed in the first week following October 7 were associated with an increased risk of PTSD. Furthermore, using information from smartwatches and daily questionnaires, we identified early markers of PTSD. The stronger the acute stress reactivity to the event was, as manifested in a sharper increase in reported stress and a sharper decrease in reported mood, step count, and sleep quality and duration) the more likely the individual was to develop PTSD symptoms.

The DSM-5’s inclusion of indirect exposure to trauma as a criterion for PTSD acknowledges the reality of ‘secondary traumatic stress’, a condition prevalent among professionals regularly exposed to the aftermath of traumatic events, such as first responders and mental health workers, and among those learning of the violent actual or threatened death of a family member or friend [29]. Highly visual social media platforms, including Telegram, Instagram, and TikTok, are gaining popularity, particularly among younger demographics [30]. Our study emphasizes the profound impact that mass media’s graphic portrayals of trauma can have on public mental health in terror and war contexts that include entire populations in a position of vulnerability, highlighting the public health importance of developing guidelines and recommendations for posting and watching such unfiltered content. The purpose of terrorism is to expand the domain of those who perceive themselves as being in danger. Given the ubiquity of video and still photography on smartphones and other portable cameras, people are now widely exposed to real images and sounds of violence, murder, sexual assault, and other trauma as it occurs.

Importantly, people are not passive consumers of such content. As suggested by Relihan et al. [31]: “When an individual or group trauma becomes a shared public experience through widespread media coverage (e.g., mass violence, being publicly outed), sharing a social identity with a targeted individual or group of victims may amplify feelings of personal vulnerability. This heightened perceived threat may draw people to engage with trauma-related media because of increased vigilance for self-relevant threats, which can, in turn, amplify distress.” Unfortunately, such exposure, whether passive or active, produces post-traumatic stress symptoms that are comparable to those that follow current Criterion A exposure [32]. This raises the question of whether the DSM’s exposure criteria should be further expanded to include seeing such images electronically and outside the context of work or personal connection to the victims.

The definition of traumatic events within the DSM has been subject to considerable debate, as highlighted by the concepts of “conceptual bracket creep” [33] and “concept creep” [34]. Since the introduction of PTSD in DSM-III (1980), the range of experiences considered traumatic has broadened significantly, with indirect experiences added in DSM-III-R (1987). While this expansion reflects a recognition of the diverse ways individuals experience trauma, it also raises legitimate concerns about undermining resilience and diluting the clinical concept of trauma. We acknowledge the importance of maintaining distinctions between degrees of trauma — there is a fundamental difference between being directly subjected to horrific experiences (e.g., being captured and tortured) and watching such events online without direct involvement. Nonetheless, it is equally important to acknowledge that indirect exposure—especially when it taps into personal or collective identity—can still lead to substantial psychological impact. Thus, while maintaining clear distinctions in clinical diagnosis, public health frameworks should also account for the broader implications of indirect trauma exposure on mental health and well-being.

While retrospective bias could influence reporting of media consumption, our findings are consistent with earlier data from studies of response to the 9/11, Boston or Paris terrorist attacks which found that early attention to media reports was similarly associated with later distress [11]. In line with [35], we argue that the DSM’s strict trauma definitions, while aiding in identifying the most affected individuals, may not comprehensively serve public health needs. An updated, context-inclusive definition of trauma and exposure is essential for addressing the broader implications of these experiences on population health.

Few studies have explored the potential of physiological data from smartwatches in assessing individual responses during the immediate aftermath of trauma. A recent study found that monitoring such biometrics could predict adverse posttraumatic neuropsychiatric sequelae following traumatic stress exposure in hospital settings [36]. Another study demonstrated the efficacy of detecting hyperarousal through heart rate and body acceleration data, achieving an 83% success rate in predicting PTSD onset in veterans [37]. Our study expands this knowledge by revealing that civilians indirectly exposed to secondary traumatic stress via mass media suffer from similar types of physiological changes in stress, sleep, and physical activity during the week after such exposure and are at increased risk of developing PTSD.

Our findings extend current knowledge linking heightened stress levels, decreased physical activity, and sleep disturbances to the development of PTSD [38–43]. Further, our findings suggest that the extent of these physiological reactions, as detected objectively via smartwatches and subjectively by the individual, provides early signs from the impact phase. The significance of these findings is amplified by the fact that a substantial number of individuals with PTSD remain undiagnosed, despite the availability of early and personalized treatments [44–48]. The absence of proper diagnosis and subsequent treatments can lead to more severe long-term health outcomes, including persistent depressive symptoms, increased suicide attempts, higher morbidity, and premature death [48–50]. The ability to monitor and analyze biometric data from smartwatches opens new avenues for identifying early signs of PTSD at the population level, particularly in cases of indirect exposure to trauma through mass media. This is crucial for timely intervention and the prevention of long-term adverse mental health outcomes.

Our study has several limitations. We assumed that the traumatic event is defined by the October 7 atrocities, but the context of our PTSD prevalence estimates includes the onset of war following the October 7 events in the Gaza Strip. However, as opposed to the ongoing trauma of the civilians in Gaza, we believe that the trauma experienced by Israeli citizens primarily stems from the specific incidents on October 7. Second, though previously validated in several settings, including in Israel [17,51], our prevalence estimate for PTSD is based on the PCL-5 survey, while a gold standard diagnosis requires assessment by healthcare professionals. Accordingly, the reported prevalence estimates may deviate from the actual prevalence, potentially resulting in either overestimation or underestimation. Third, although the panel study sample is representative of the adult Israeli Jewish population in terms of age, sex, and geographical location, individuals more profoundly impacted by the traumatic event may have been disproportionately inclined to participate. This potential selection bias should be considered when interpreting the findings. Another limitation is that we did not incorporate broader mental health screening tools like the PHQ-9 for depression. While this decision was driven by the need to minimize participant burden during an acute crisis, it limits our understanding of how pre-existing or concurrent mood disorders might influence PTSD development and persistence. Future research should consider incorporating additional validated screening tools to understand better the bidirectional relationship between depression, mood disorders and PTSD, particularly given their overlapping symptomatology and frequent co-occurrence. We also note that in our analysis of the relationship between indirect exposure to trauma and PTSD, we relied on self-reported measures—namely, the frequency of news consumption and the number of gory videos viewed—which are subject to recall bias and individual interpretation. Finally, the lack of longitudinal data limits our understanding of PTSD’s progression, severity, and long-term impacts. Future research should include longer follow-up periods to provide a more comprehensive understanding of the trajectory of PTSD over time.

In conclusion, our findings demonstrate that PTSD rates among those indirectly exposed to mass traumatic events through media exposure can reach unprecedented levels, with prevalence remaining exceptionally high even seven months after the event. These results underscore both the profound psychological impact of indirect trauma exposure through social media and other indirect outlets and the potential of wearable technology to enable early identification of individuals at risk for PTSD, potentially creating new opportunities for timely intervention and prevention.

## Supporting information

S1 Appendix Study Protocol, data collection platform and data access, and prospective study participants’ adherence. (DOCX)

S2 Appendix The online survey. (DOCX)

S3 Appendix Statistical analysis. (DOCX)

S4 Appendix Additional results. (DOCX)

## References

[1] Ressler KJ, Berretta S, Bolshakov VY, Rosso IM, Meloni EG, Rauch SL, et al. Post-traumatic stress disorder: clinical and translational neuroscience from cells to circuits. Nat Rev Neurol. 2022;18(5):273–88. doi: 10.1038/s41582-022-00635-8 35352034 PMC9682920

[2] Holman EA, Garfin DR, Lubens P, Silver RC. Media exposure to collective trauma, mental health, and functioning: Does it matter what you see? Clinical Psychological Science. 2020;8:111–24. doi: 10.1177/2167702619858300

[3] Robert M, Stene LE, Garfin DR, Vandentorren S, Motreff Y, du Roscoat E, et al. Media Exposure and Post-traumatic Stress Symptoms in the Wake of the November 2015 Paris Terrorist Attacks: A Population-Based Study in France. Front Psychiatry. 2021;12:509457. doi: 10.3389/fpsyt.2021.509457 34093248 PMC8175798

[4] Galea S, Vlahov D, Resnick H, Ahern J, Susser E, Gold J, et al. Trends of probable post-traumatic stress disorder in New York City after the September 11 terrorist attacks. Am J Epidemiol. 2003;158(6):514–24. doi: 10.1093/aje/kwg187 12965877

[5] Neria Y, Sullivan GM. Understanding the mental health effects of indirect exposure to mass trauma through the media. JAMA. 2011;306(12):1374–5. doi: 10.1001/jama.2011.1358 21903818 PMC3637659

[6] Column: Israel’s Darkest Hour Casts a Shadow on the World | TIME. [cited 15 Dec 2023]. Available: https://time.com/6321967/israel-president-isaac-herzog-hamas-attack/

[7] Bryant RA. A critical review of mechanisms of adaptation to trauma: Implications for early interventions for posttraumatic stress disorder. Clin Psychol Rev. 2021;85:101981. doi: 10.1016/j.cpr.2021.101981 33588312

[8] McEwen BS, Seeman T. Protective and damaging effects of mediators of stress. Elaborating and testing the concepts of allostasis and allostatic load. Ann N Y Acad Sci. 1999;896:30–47. doi: 10.1111/j.1749-6632.1999.tb08103.x 10681886

[9] Akil H, Nestler EJ. The neurobiology of stress: Vulnerability, resilience, and major depression. Proc Natl Acad Sci U S A. 2023;120(49):e2312662120. doi: 10.1073/pnas.2312662120 38011574 PMC10710064

[10] Bryant RA, Creamer M, O’Donnell M, Forbes D, McFarlane AC, Silove D, et al. Acute and Chronic Posttraumatic Stress Symptoms in the Emergence of Posttraumatic Stress Disorder: A Network Analysis. JAMA Psychiatry. 2017;74(2):135–42. doi: 10.1001/jamapsychiatry.2016.3470 28002832

[11] Butler LD, Koopman C, Azarow J, Blasey CM, Magdalene JC, DiMiceli S, et al. Psychosocial predictors of resilience after the September 11, 2001 terrorist attacks. J Nerv Ment Dis. 2009;197(4):266–73. doi: 10.1097/NMD.0b013e31819d9334 19363383

[12] Mofaz M, Yechezkel M, Einat H, Kronfeld-Schor N, Yamin D, Shmueli E. Real-time sensing of war’s effects on wellbeing with smartphones and smartwatches. Commun Med (Lond). 2023;3(1):55. doi: 10.1038/s43856-023-00284-y 37069232 PMC10109229

[13] Yechezkel M, Mofaz M, Painsky A, Patalon T, Gazit S, Shmueli E, et al. Safety of the fourth COVID-19 BNT162b2 mRNA (second booster) vaccine: a prospective and retrospective cohort study. Lancet Respir Med. 2023;11(2):139–50. doi: 10.1016/S2213-2600(22)00407-6 36410364 PMC9889528

[14] Mofaz M, Yechezkel M, Guan G, Brandeau ML, Patalon T, Gazit S, et al. Self-Reported and Physiologic Reactions to Third BNT162b2 mRNA COVID-19 (Booster) Vaccine Dose. Emerg Infect Dis. 2022;28(7):1375–83. doi: 10.3201/eid2807.212330 35654410 PMC9239876

[15] Guan G, Mofaz M, Qian G, Patalon T, Shmueli E, Yamin D, et al. Higher sensitivity monitoring of reactions to COVID-19 vaccination using smartwatches. NPJ Digit Med. 2022;5(1):140. doi: 10.1038/s41746-022-00683-w 36085312 PMC9461410

[16] Oved S, Mofaz M, Lan A, Einat H, Kronfeld-Schor N, Yamin D, et al. Differential effects of COVID-19 lockdowns on well-being: interaction between age, gender and chronotype. J R Soc Interface. 2021;18(179):20210078. doi: 10.1098/rsif.2021.0078 34062107 PMC8169206

[17] Blevins CA, Weathers FW, Davis MT, Witte TK, Domino JL. The Posttraumatic Stress Disorder Checklist for DSM-5 (PCL-5): Development and Initial Psychometric Evaluation. J Trauma Stress. 2015;28(6):489–98. doi: 10.1002/jts.22059 26606250

[18] Löwe B, Decker O, Müller S, Brähler E, Schellberg D, Herzog W, et al. Validation and standardization of the Generalized Anxiety Disorder Screener (GAD-7) in the general population. Med Care. 2008;46(3):266–74. doi: 10.1097/MLR.0b013e318160d093 18388841

[19] Gepner Y, Mofaz M, Oved S, Yechezkel M, Constantini K, Goldstein N, et al. Utilizing wearable sensors for continuous and highly-sensitive monitoring of reactions to the BNT162b2 mRNA COVID-19 vaccine. Commun Med (Lond). 2022;2:27. doi: 10.1038/s43856-022-00090-y 35603274 PMC9053261

[20] VÍVOSMART ® 4 Owner’s Manual. 2018.

[21] Sleep Problems and PTSD - PTSD: National Center for PTSD. [cited 15 Dec 2023]. Available: https://www.ptsd.va.gov/understand/related/sleep_problems.asp

[22] Zen AL, Whooley MA, Zhao S, Cohen BE. Post-traumatic stress disorder is associated with poor health behaviors: findings from the heart and soul study. Health Psychol. 2012;31(2):194–201. doi: 10.1037/a0025989 22023435 PMC3295904

[23] ISTSS - Posttraumatic Stress Disorder Checklist. [cited 15 Dec 2023]. Available: https://istss.org/clinical-resources/assessing-trauma/ptsd-checklist-dsm-5?gclid=Cj0KCQiAgqGrBhDtARIsAM5s0_kIFlV4y3jn36yr2sC5-6VohlDk05TMyRsIosd5YiZObB7N1DWkRMgaAsJnEALw_wcB

[24] COVID-19 Datasets- Government Data. [cited 30 May 2020]. Available: https://data.gov.il/dataset/covid-19

[25] Galea S, Ahern J, Resnick H, Kilpatrick D, Bucuvalas M, Gold J, et al. Psychological sequelae of the September 11 terrorist attacks in New York City. N Engl J Med. 2002;346(13):982–7. doi: 10.1056/NEJMsa013404 11919308

[26] May JM, Richardi TM, Barth KS. Dialectical behavior therapy as treatment for borderline personality disorder. Ment Health Clin. 2016;6(2):62–7. doi: 10.9740/mhc.2016.03.62 29955449 PMC6007584

[27] Figley CR. Compassion fatigue as secondary traumatic stress disorder: An overview. Figley CR, editor. Compassion fatigue: Coping with secondary traumatic stress disorder in those who treat the traumatised. New York: Brunner-Routledge; 1995. Available: http://books.google.com/books?hl=en&lr=&id=2Cwo47uOEq4C&pgis=1

[28] Bride BE. Prevalence of secondary traumatic stress among social workers. Soc Work. 2007;52(1):63–70. doi: 10.1093/sw/52.1.63 17388084

[29] American Psychiatric Association. Diagnostic and Statistical Manual of Mental Disorders. Diagnostic and Statistical Manual of Mental Disorders. 2022. 10.1176/APPI.BOOKS.9780890425787

[30] McCrory E, Foulkes L, Viding E. Social thinning and stress generation after childhood maltreatment: a neurocognitive social transactional model of psychiatric vulnerability. Lancet Psychiatry. 2022;9(10):828–37. doi: 10.1016/S2215-0366(22)00202-4 35926524

[31] Relihan DP, Jones NM, Holman EA, Silver RC. Shared social identity and media transmission of trauma. Sci Rep. 2023;13(1). doi: 10.1038/s41598-023-33898-2 PMC10354080 37463937

[32] Howard J, Lorenzo-Luaces L, Lind C, Lakhan P, Rutter LA. Is a Criterion A trauma necessary to elicit posttraumatic stress symptoms? J Psychiatr Res. 2024;170:58–64. doi: 10.1016/j.jpsychires.2023.12.008 38103450

[33] McNally RJ. Remembering trauma Richard J. McNally. Belknap Press of Harvard University Press; 2005.

[34] Haslam N. Concept Creep: Psychology’s Expanding Concepts of Harm and Pathology. Psychological Inquiry. 2016;27(1):1–17. doi: 10.1080/1047840x.2016.1082418

[35] Gradus JL, Galea S. Moving From Traumatic Events to Traumatic Experiences in the Study of Traumatic Psychopathology. Am J Epidemiol. 2023;192(10):1609–12. doi: 10.1093/aje/kwad126 37218615 PMC10558183

[36] Straus LD, An X, Ji Y, McLean SA, Neylan TC, AURORA Study Group, et al. Utility of Wrist-Wearable Data for Assessing Pain, Sleep, and Anxiety Outcomes After Traumatic Stress Exposure. JAMA Psychiatry. 2023;80(3):220–9. doi: 10.1001/jamapsychiatry.2022.4533 36630119 PMC9857758

[37] Sadeghi M, McDonald AD, Sasangohar F. Posttraumatic stress disorder hyperarousal event detection using smartwatch physiological and activity data. PLoS One. 2022;17(5):e0267749. doi: 10.1371/journal.pone.0267749 35584096 PMC9116643

[38] Zhang Y, Ren R, Sanford LD, Yang L, Zhou J, Zhang J, et al. Sleep in posttraumatic stress disorder: A systematic review and meta-analysis of polysomnographic findings. Sleep Med Rev. 2019;48:101210. doi: 10.1016/j.smrv.2019.08.004 31518950

[39] Gehrman P, Seelig AD, Jacobson IG, Boyko EJ, Hooper TI, Gackstetter GD, et al. Predeployment Sleep Duration and Insomnia Symptoms as Risk Factors for New-Onset Mental Health Disorders Following Military Deployment. Sleep. 2013;36(7):1009–18. doi: 10.5665/sleep.2798 23814337 PMC3669076

[40] Hegberg NJ, Hayes JP, Hayes SM. Exercise Intervention in PTSD: A Narrative Review and Rationale for Implementation. Front Psychiatry. 2019;10:133. doi: 10.3389/fpsyt.2019.00133 30949075 PMC6437073

[41] Whitworth JW, Ciccolo JT. Exercise and Post-Traumatic Stress Disorder in Military Veterans: A Systematic Review. Mil Med. 2016;181(9):953–60. doi: 10.7205/MILMED-D-15-00488 27612337

[42] Vancampfort D, Stubbs B, Richards J, Ward PB, Firth J, Schuch FB, et al. Physical fitness in people with posttraumatic stress disorder: a systematic review. Disabil Rehabil. 2017;39(24):2461–7. doi: 10.1080/09638288.2016.1226412 27628485

[43] Miller KE, Rasmussen A. War exposure, daily stressors, and mental health in conflict and post-conflict settings: bridging the divide between trauma-focused and psychosocial frameworks. Soc Sci Med. 2010;70(1):7–16. doi: 10.1016/j.socscimed.2009.09.029 19854552

[44] Smith Flowers P, Larkin MJ. Interpretative phenomenological analysis: Theory, method and research. Thousand Oaks, CA: Sage Publications Inc; 2009; 225. Available: https://www.researchgate.net/publication/221670349_Interpretative_Phenomenological_Analysis_Theory_Method_and_Research

[45] Grinage BD. Diagnosis and management of post-traumatic stress disorder. Am Fam Physician. 2003;68(12):2401–8. 14705759

[46] Meltzer LJ, Montgomery-Downs HE, Insana SP, Walsh CM. Use of actigraphy for assessment in pediatric sleep research. Sleep Med Rev. 2012;16(5):463–75. doi: 10.1016/j.smrv.2011.10.002 22424706 PMC3445439

[47] Lecrubier Y. Posttraumatic Stress Disorder in Primary Care: A Hidden Diagnosis. J Clin Psychiatry. 2004;65:14894. 14728097

[48] Gagnon-Sanschagrin P, Schein J, Urganus A, Serra E, Liang Y, Musingarimi P, et al. Identifying individuals with undiagnosed post-traumatic stress disorder in a large United States civilian population - a machine learning approach. BMC Psychiatry. 2022;22(1):630. doi: 10.1186/s12888-022-04267-6 36171558 PMC9519190

[49] Goenjian AK, Walling D, Steinberg AM, Karayan I, Najarian LM, Pynoos R. A prospective study of posttraumatic stress and depressive reactions among treated and untreated adolescents 5 years after a catastrophic disaster. Am J Psychiatry. 2005;162(12):2302–8. doi: 10.1176/appi.ajp.162.12.2302 16330594

[50] Priebe S, Katsakou C, Amos T, Leese M, Morriss R, Rose D, et al. Patients’ views and readmissions 1 year after involuntary hospitalisation. Br J Psychiatry. 2009;194(1):49–54. doi: 10.1192/bjp.bp.108.052266 19118325

[51] Raz A, Rubinstein R, Shadach E, Chaikin G, Ben Yehuda A, Tatsa-Laur L, et al. Behavioral Self-Blame in PTSD—Etiology, Risk Factors, and Proposed Interventions. IJERPH. 2023;20(15):6530. doi: 10.3390/ijerph20156530 37569070 PMC10418798

