# Supplementary material for: Social media impact and smartwatch monitoring: Prevalence and early markers of PTSD and anxiety following mass traumatic events
Source: PLOS Ment Health. 2025 Sep 10;2(9):e0000195. doi: 10.1371/journal.pmen.0000195 (PMC12798574; doi:10.1371/journal.pmen.0000195)
Supplement: S1 Appendix — (DOCX) [file pmen.0000195.s001.docx]

S1 Appendix for:

**Social Media Impact and Smartwatch Monitoring: Prevalence and Early Markers of PTSD and Anxiety Following Mass Traumatic Events**

Dan Yamin^1,2,3,†^, Shahar Lev-Ari^4,†^, Merav Mofaz^1^, Ron Elias^1^, Sharon Toker^5^, David Spiegel^6^, Matan Yechezkel^1^, Margaret L. Brandeau^2^, Erez Shmueli^1,3,7,†,*^

^1^ Department of Industrial Engineering, Tel Aviv University, Tel Aviv, Israel.

^2^ Department of Management Science and Engineering, Stanford University, Stanford, California, United States of America.

^3^ Wizermed D.H. LTD, Zoran, Israel.

^4^ Department of Health Promotion, Tel Aviv University, Tel Aviv, Israel.

^5^ Coller School of Management, Tel Aviv University, Tel Aviv, Israel.

^6^ Department of Psychiatry and Behavioral Sciences, Stanford University, Stanford, California, United States of America.

^7^ MIT Media Lab, MIT, Cambridge, Massachusetts, United States of America.

^†^ Contributed equally.

^*^ [shmueli@tau.ac.il](mailto:shmueli@tau.ac.il)

# **Study protocol**

# Prospective part

***Study Design***

In this study we will analyze data that were already collected and will be collected as part of the PerMed study [1]. Participants in the PerMed study are recruited for a period of two years, during which they are equipped with a Garmin Vivosmart 4 smartwatches and are asked to wear them as much as they could. In addition, participants install two applications on their mobile phones: an application that passively collects data from the smartwatch and a dedicated mobile application which allows participants to fill a daily questionnaire. We will also ask active participants to fill in dedicated surveys for the detection of Post-Traumatic Stress Disorder (PTSD) and anxiety. In this study, we will consider for each participant the week prior to October 7, 2023, as the baseline period.

***Participants***

The inclusion criteria for the PerMed study includes those aged > 18 years. Individuals who are not eligible to give and sign a consent form are excluded. In this study, we will analyze the data of participants aged 18 years and above before and after October, 7, 2023. To recruit participants and ensure they complete all the study’s requirements, we will hire a professional survey company. Potential participants will be recruited through advertisements in social media, online banners, and word-of-mouth. The survey company is responsible for guaranteeing the participants meet the study’s requirements, in particular, that the questionnaires are filled daily, ensuring the smartwatches are charged constantly and worn properly, and assisting participants resolve technical problems.

***Study procedures***

Before participation in the study, all participants will be advised orally and in writing about the nature of the experiments and give written, informed consent. At this time, participants will be asked to complete an enrollment questionnaire that includes demographic information and health status. In addition, participants will be asked to install two applications on their mobile phones: an application that passively collects data from the smartwatch and the PerMed application, which allows participants to fill in the daily questionnaires. Participants will be given instructions regarding the self-reported symptoms questionnaires and how to operate the smartwatch, which they will wear as much as they can. We will also ask active participants to fill in dedicated surveys of PTSD eight weeks after the events of October 7, 2023 to detect possible signs of PTSD.

***Enrollment questionnaire***

All participants will fill in a one-time enrollment questionnaire that includes demographic questions and questions about the participant’s health condition in general. Specifically, the questionnaire will include the following: age, sex, height, weight, underlying medical conditions, and household income (Listed in Table S2). Other questions such as name, address, phone and email will be recorded and used by the survey company to contact the participants. The answers will be filled-in directly by the survey company to the study’s secured dashboard.

***Monitoring device***

Participants will be equipped with Garmin Vivosmart 4 smart fitness trackers. Among other features, the smartwatch provides all-day heart rate and heart rate variability and during-night blood oxygen saturation level tracking capabilities [2].

The optical wrist heart rate (HR) monitor of the smartwatch is designed to continuously monitor a user’s heart rate. The frequency at which heart rate is measured varies and may depend on the level of activity of the user: when the user starts an activity, the optical HR monitor’s measurement frequency increases.

Since heart rate variability (HRV) is not easily accessible through Garmin’s application programming interface (API), we use Garmin’s stress level instead, which is calculated based on HRV. Specifically, the device uses heart rate data to determine the interval between each heartbeat. The variable length of time between each heartbeat is regulated by the body's autonomic nervous system. Less variability between beats correlates with higher stress levels, whereas an increase in variability indicates less stress [3]. A similar relationship between HRV and stress was also seen in [4,5].

Examining the data collected in our study, we identified an HR sample roughly every 15 seconds, a stress – based HRV sample every 180 seconds.

While the Garmin smartwatch provides state-of-the-art wrist monitoring, it is not a medical-grade device, and some readings may be inaccurate under certain circumstances, depending on factors such as the fit of the device and the type and intensity of the activity undertaken by a participant [6–8].

***Daily questionnaires***

All participants will complete the daily self-reported questionnaire in a dedicated application (the PerMed mobile application). The daily questionnaire we will use includes the following questions:

| 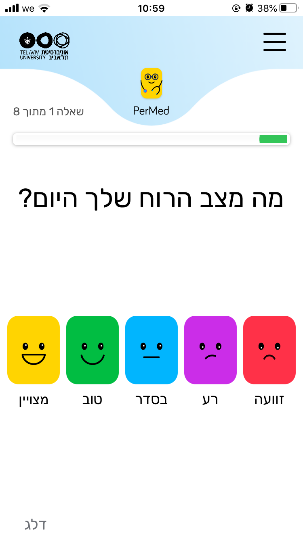 | How is your mood today? • Awful (1)• Bad (2)• OK (3)• Good (4)• Excellent (5) |
| --- | --- |
| 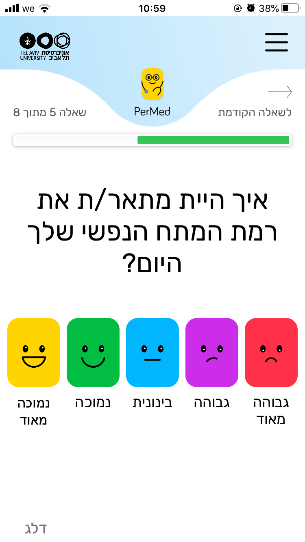 | How would you describe the level of your stress during the last day?• Very Low (1)• Low (2)• Medium (3)• High (4)• Very high (5) |
| 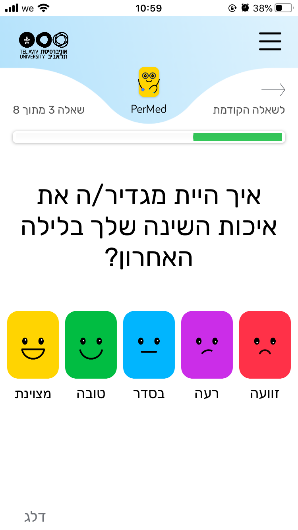 | How would you define your last night sleep quality?• Awful (1)• Bad (2)• OK (3)• Good (4)• Excellent (5) |
| 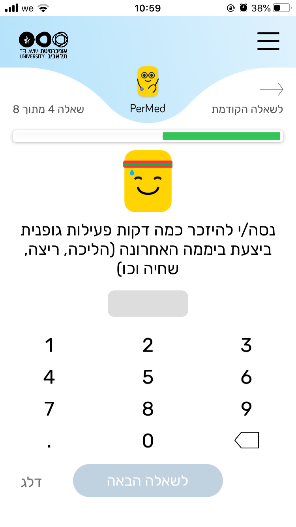 | Try to remember how many minutes of sports activity you performed on the last day? |
| 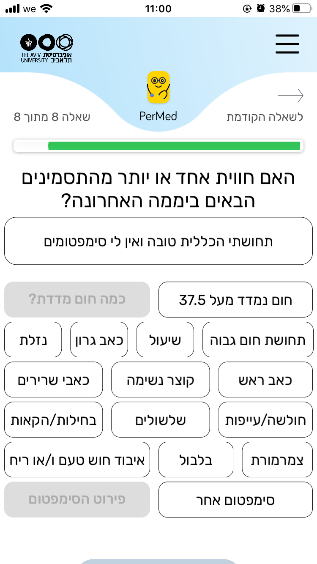 | Have you experienced one or more of the following symptoms in the last 24 hours?• My general feeling is good, and I have no symptoms• Heat measured above 37.5• Cough• Sore throat• Runny nose• Headache• Shortness of breath• Muscle aches• Weakness / fatigue• Diarrhea• Nausea / vomiting• Chills• Confusion• Loss of sense of taste / smell• Another symptom. |

***PTSD dedicated survey***

All participants will be asked to fill in an online survey that includes well-established PTSD surveys via the PerMed application. The Posttraumatic Stress Disorder Checklist (PCL-5) is a self-report questionnaire designed to assess the 20 symptoms outlined in the DSM-5 criteria for PTSD. The PCL-5 serves various purposes, including screening individuals for PTSD and making provisional diagnoses [9]. It is widely used for clinical and research purposes; psychometrically, the PCL-5 demonstrates strong internal consistency (α = .94), high test-retest reliability (r = .82), and strong convergent validity (rs = .74 to .85) [9]. We will utilize the General Anxiety Disorder (GAD) 7 item questionnaire (GAD-7) to identify cases of GAD along with measuring anxiety symptom severity. The tool is also widely used as a screening measure of panic, social anxiety, and PTSD. The questionnaire is considered valid, sensitive, and specific for the diagnosis of GAD in the general population [10]. Will use the Israeli Ministry of Health’s (IMOH) Hebrew translation of these surveys. See S2 Appendix for the full survey.

***Data Storage***

Data collected from the mobile phone application and from the smartwatches will be stored on a secure server within Tel Aviv University facilities. The server runs a CentOS operating system and is located in the Software Engineering Building at Tel Aviv University. This server is protected behind the university's firewall and is not connected to external networks. In addition, a secure connection through an SSL protocol and a trusted certificate will be obtained for the transfer of information from the mobile phone application into the secured server.

Access will be restricted to investigators in the study. The information from the mobile application will be stored in a structured manner on the secured server without any explicitly identifying information (name, ID number, email). Each participant will be assigned a coded participant number that will be used to identify the subject in the database. The code with the identified information will be stored in an encrypted form on a separate secured server that only the research manager will have access to. Access to all servers is restricted with username and password.

All (non-digital) questionnaires and signed informed consent documents will be stored in a secured cabinet in Tel Aviv University, to which only the research manager and the principal investigators will have access. No data collected as part of the study will be added to individuals’ medical charts.

***Data preprocessing***

We will perform several preprocessing steps. Concerning the daily questionnaires, in cases where participants will fill in the daily questionnaire more than once on a given day, only the last entry for that day will be considered, as it is reasoned that the last one likely best represented the entire day. Self-reported symptoms that are entered as the free text will be manually categorized.

A participant that completed a PTSD survey will also be categorized with PTSD if the total score of the PCL-5 questionnaire is $\geq$ 33 and meet the DSM-5 diagnostic rule which requires at least: 1 B item (questions 1-5), 1 C item (questions 6-7), 2 D items (questions 8-14), 2 E items (questions 15-20). In line with the GAD-7 criteria, anxiety level of participant will be determined by the total score: no anxiety (0-4), mild (5-9), moderate (10-14) and severe ($\geq$15). Then we will compute the proportion of participants presenting PTSD and proportions of each anxiety level.

***Data Analysis and inclusion criteria***

For a more comprehensive understanding of individual perception, we will examine the reported stress levels throughout the PerMed study period. We will calculate the daily centered moving average and the associated 95% confidence interval with a 7-day window from January 1, 2021, for all active participants. For participants who filled in the PTSD online survey, we will implement the same procedure, stratifying them based on whether they exhibited PTSD.

For each participant who will remain active after October 7, 2023, we will assess the reported sleep quality, as well as various physiological indicators: duration of awake time during sleep, step counts, and duration of high stress levels. We will compute a single weighted average value for each of the two periods — a week prior to October 7, 2023 (baseline period) and the week after October 7, 2023. For each indicator, we will utilize paired t-test to examine the difference between the weighted averages during the baseline period and those following October 7, 2023.

The weighted average for each participant will be determined by first averaging the corresponding indicator values separately for workdays and free days (weekends and national holidays). Subsequently, the weighted average of these two values will be calculated, assigning a weight of 5/7 to workdays and 2/7 to free days. The rationale for computing this weighted average is further explained in [11]. In essence, we identified a weekly rhythm across various indicators, with free days exhibiting different mean daily values than workdays. Given the relatively short duration of the examined time periods and variations in the number of free days (e.g., holidays), we aimed to correct for potential biases. Finally, we will calculate the mean and its corresponding 95% confidence interval for each indicator across the participants.

For participants who filled in the PTSD online survey, we will repeat the above analysis, by stratifying the participants based on whether they exhibited PTSD.

For each of the well-being indicators, we will explore the associations between the probability of PTSD, the weighted average during the baseline period, and the difference in the weighted average between baseline and the week after October 7 periods, while controlling for other explanatory variables (age and sex).

All statistical analyses will be performed using Python 3.8.

***Potential Risks & Risk management***

No specific risks arising from the smartwatches are expected, as the device is already commercialized with no known adverse reactions. The main risk in this study is the leakage of private data which we intend to manage as we describe in the following section.

***Privacy/Confidentiality***

Results from this study will be handled at an aggregated level. Individual data records will remain confidential and will not be published or shared with any third party. Signed and dated informed consent forms, as well as data recording sheets (e.g., case report forms) will be stored in locked cabinets during the study and following its completion. A file containing the personal details of the participants will be coded to help preserve confidentiality and will be separated from all other data collected throughout the study. This file will be kept by the principal investigator. Data will be stored on computers in password-protected files.

The data obtained from the smartwatch used in this study will be linked to a coded participant number. The smartwatch does not include a GPS. The data collected by the PerMed application will arrive directly to PerMed back-end servers and will be stored securely.

# Panel study

***Study Design***

In this study, we will recruit and analyze data of 2,500 participants, forming a representative sample of the Jewish Israeli population in terms of age, sex, and geographical location. Participants will be asked to fill in online dedicated surveys for the detection of PTSD and anxiety. Participants will also be asked to provide basic demographic data such as age, sex, educational background, place of residence, etc.

***Participants***

Inclusion criteria include individuals age > 18 years from the Jewish Israeli population. To recruit participants for the panel study and ensure they complete all the study’s requirements, we will hire a professional survey company. Potential participants will be recruited through a preregistered panelists' database via email. The survey company will ensure that the participants are representative of the Israeli population in term of age, sex and geographical location. To incentivize participants to complete all study’s requirements (i.e., complete the PTSD survey), each participant will receive 3 New Israeli Shekels upon completion of study’s requirements.

***Study procedures***

Before participating in the study, all participants will be advised in writing about the nature of the survey (see S2 Appendix). At this time, participants will be asked to complete the PTSD online survey about coping with the events that began on October 7, that includes demographic information and news consumption behavior, to detect a possible sign of PTSD nationwide Participants will also be invited to complete the same PTSD online survey seven months after October 7, 2023, for follow-up assessment.

***Description of the data***

Data that will be collected in the online survey includes (see the survey questions in S2 Appendix):

- Sociodemographic:
  - Sex
  - Age
  - Household size
  - Household income
  - Religion
  - Place of residence (city name)
- History of PTSD and anxiety (binary)
- News consumption behavior
  - News consumption frequency
  - News consumption platforms
- PCL-5 and GAD-7 questionnaire

***Data collection and storage***

The data will be extracted and stored on a secure server within Tel Aviv University facilities. The server runs a CentOS operating system and is located in the Software Engineering Building at Tel Aviv University. This server is protected behind the university's firewall and is not connected to external networks.

Access will be restricted to investigators in the study. The information from the online PTSD survey will be stored in a structured manner on the secured server without any explicitly identifying information (name, ID number, email). Each participant will be assigned a coded participant number that will be used to identify the subject in the database.

***Data Analysis and inclusion criteria***

The questionnaire data will be preprocessed by manually categorizing any self-reported symptom of PTSD and anxiety according to the PCL-5 and GAD-7 criteria. Participant will be categorized as PTSD if the total score of the PCL-5 questionnaire is $\geq$ 33 and meet the DSM-5 diagnostic rule which requires at least: 1 B item (questions 1-5), 1 C item (questions 6-7), 2 D items (questions 8-14), 2 E items (questions 15-20). In line with the GAD-7 criteria, anxiety level of participant will be determined by the total scores: no anxiety (0-4), mild (5-9), moderate (10-14) and severe ($\geq$15).

We will analyze the data of participants who had either direct or indirect exposure to the events of October 7. We define direct exposure as participants who were evacuated from their place of residence or indicated that they or their immediate family were injured, killed or abducted. Indirect exposure refers to all other cases. For each level of exposure, we will evaluate the prevalence estimate of PTSD as the proportions of PTSD (as assessed in the first PTSD survey). we will also stratify the prevalence estimate by the duration of news information consumption during the first week after October 7 and the extent of exposure to gory videos. Additionally, we will examine the rates of each anxiety level.

We will also evaluate the rates of exposure to gory videos on each media platform. Specifically, we will divide the number of participants who were exposed to gory videos on each platform by the number of participants who consume news information using that platform.

The 95% confidence intervals (CIs) will be obtained under the assumption of binomial distribution.

To examine the relation between the probability of PTSD (as indicated by the first survey) and news information consumption behavior, controlling for other explanatory variables (age, sex, educational background, PTSD background, religious level, and socioeconomic level), we will fit a logistic regression model.

Likewise, we will also examine the associations between the probability of moderate to severe anxiety, duration of news consumption during the two weeks before filling in the first online PTSD survey, and the extent of exposure to gory videos, while controlling for other explanatory variables (age, sex, educational background, anxiety background, religious level, and socioeconomic level).

***Potential Risks & Risk management***

The main risk in this study is the leakage of private data which we intend to manage as we describe in the following section.

***Privacy/Confidentiality***

Results from this study will be handled at an aggregated level. Individual data records will remain confidential and will not be published or shared with any third party.

# **Data collection platform and data access**

## Architecture

The data collection platform contains several components that interact with each other (see Fig S1.1):

- **The PerMed application** – This application is installed on each participant’s phone to collect sensors data and the self-reported daily questionnaires. It also handles the smartwatch pairing. The current version of the application supports both Android and iOS devices.
- **The smartwatch** - send the data to the Garmin Connect app on the smartphone, which then sends these data to Garmin’s server.
- **The smartwatch application** – This application (currently Garmin) receives information from the smartwatch via Bluetooth and transmits it to the company's server. In addition, it provides a convenient interface for displaying the participant's smartwatch information.
- **The app server** – The webserver handles the database connectivity using REST API pages. It enables the server to authenticate users as they launch the application and write records to the database. A MySQL server stores the sensors' raw data and the answers to the daily questionnaires. At last, there is a batch processes running on the server that sends app notifications (daily reminder to fill the questionnaire).
- **The dashboard server** - hosts the dashboard pages, which assist in monitoring the quality of the information and controlling the experiment. The dashboard has access to participant information and signals indicating whether questionnaires were completed and the smart watch was worn without seeing its content directly. A batch process is responsible for aggregating raw data for dashboard statistics.
- **The smartwatch server** - A MySQL server stores the smartwatch data. A batch process is responsible for collecting the data from the Garmin server.

| 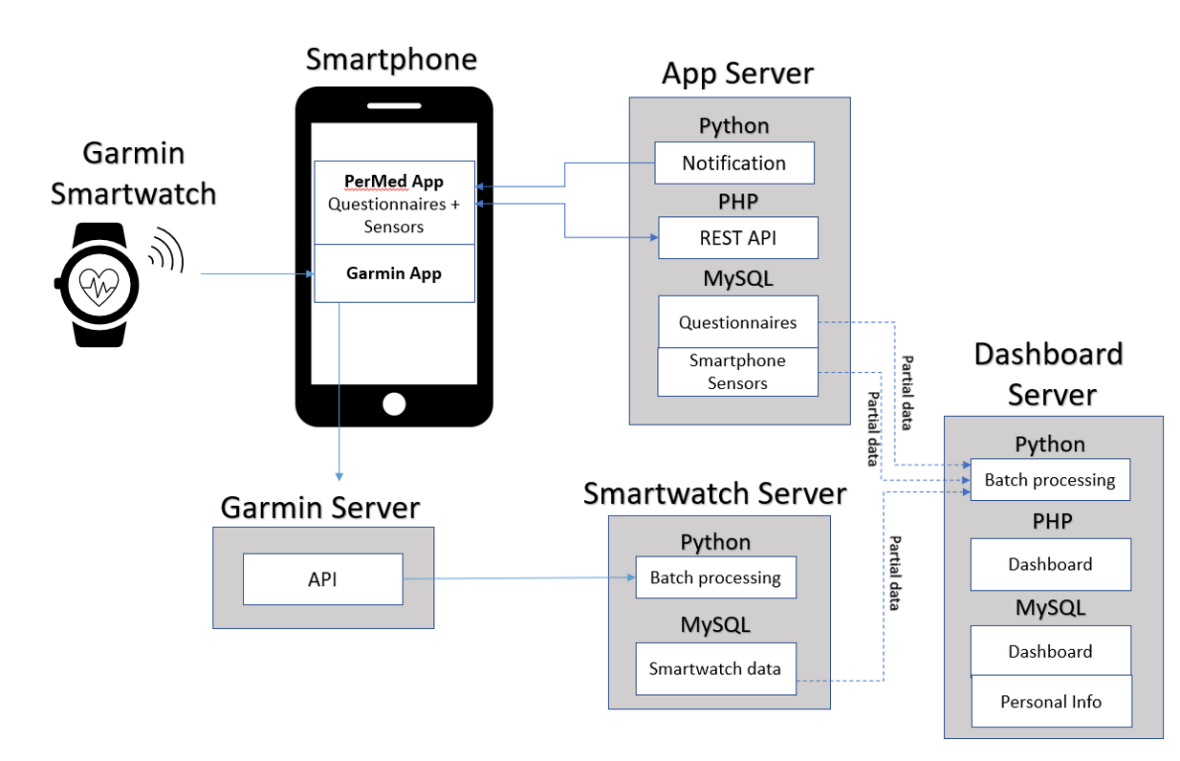 |
| --- |
| **Fig S1.1.** The high-level architecture of the PerMed’s data collection platform. |

## The PerMed Dashboard

Participants will be recruited by a qualified external recruitment team headed by Tel Aviv University personal. The team receive limited information essential control the experiment. Thus, we developed a dedicated dashboard for monitoring the quality of the information and control the experiment. This dashboard aims to identify data collection issues such as participants who did not fill the daily questionnaires or participants who did not charge the battery of their smartwatches. The dashboard also helps us identify problems that were not related to participants’ cooperation, such as bugs in the mobile app. This identification allows us to respond faster and provide timely solutions.

## The Type of Data Collected and data access

Data collected by the platform arrive from four primary sources:

- - - - **Enrollment questionnaire -** data were collected from a one-time enrollment questionnaire that includes basic personal characteristics such as socio-demographic information (e.g., age, gender, height, weight), general habits, health status, and a short Big Five personality questionnaire.
      - **Daily questionnaire** – consists of questions on 1) wellbeing, 2) general health condition, 3) symptoms observed, 4) test results to diagnose infectious diseases, 4) vaccination or medication consumption (if relevant to the study question).
      - **Smartphone sensor data** – consist of location, Wi-Fi, Bluetooth, screen, and activity.
      - **Smartwatch data** - consist of heart rate data, accelerometer and gyroscope information and measures based on these data including active minutes, steps, distance, calories, and sleep level classification, including light, deep, REM, and awake periods during sleep.

The current research, aims to explore the safety of vaccination, is part of a larger study. Raw accelerometer data, mobile activity and GPS locations are generally considered sensitive information. In accordance with the data minimization principle, we did not extract this type of data for this vaccination safety research.

# **Prospective study participants’ adherence**

We employed a professional survey company to recruit participants and ensure they adhere to the study requirements. Participant recruitment was performed via advertisements on social media and word-of-mouth. Each participant signed an informed consent form after receiving a comprehensive explanation on the study. Then, participants completed a one-time enrollment questionnaire, were equipped with Garmin Vivosmart 4 smartwatches, and installed two applications on their mobile phones: (1) the PerMed application [1,12,13], which collects daily self-reported questionnaires, and (2) an application that passively records smartwatch data. Participants were asked to wear their smartwatches as much as possible. The survey company ensured that participants' questionnaires were filled at least twice a week, that their smartwatches were charged and properly worn, and that any technical problems with the mobile applications or smartwatch were resolved. Participants were monitored through the mobile application and smartwatches for a period of at least 49 days, starting seven days before vaccination. Participants also granted full access to their EMR data.

We implemented several preventive measures to minimize participant attrition and discomfort as a means to improve the quality, continuity and reliability of the collected data. First, each day, participants who did not fill their daily questionnaire by 7 pm received a reminder notification through the PerMed application. Second, we developed a dedicated dashboard that allowed the survey company to identify participants who repeatedly neglected to complete the daily questionnaire or did not wear their smartwatch for extended periods of time; these participants were contacted by the survey company (either by text message or phone call) and encouraged to better adhere to the study protocol. Third, to strengthen participants' engagement, a weekly personalized summary report was generated for each participant, which was available inside the PerMed application. Similarly, a monthly newsletter with recent findings from the study and useful tips regarding the smartwatch's capabilities was sent to the participants. At the end of the study, participants will receive all personal insights that were obtained and can keep the smartwatch as a gift.

# **References**

1. Oved S, Mofaz M, Lan A, Einat H, Kronfeld-Schor N, Yamin D, et al. Differential effects of COVID-19 lockdowns on well-being: interaction between age, gender and chronotype. 2021 Jan. doi:10.21203/RS.3.RS-137929/V1

2. VÍVOSMART ® 4 Owner’s Manual. 2018.

3. What Is the Stress Level Feature on My Garmin Watch? | Garmin Support. [cited 24 Aug 2021]. Available: https://support.garmin.com/en-US/?faq=WT9BmhjacO4ZpxbCc0EKn9

4. Kim H-G, Cheon E-J, Bai D-S, Lee YH, Koo B-H. Stress and Heart Rate Variability: A Meta-Analysis and Review of the Literature. Psychiatry Investig. 2018;15: 235. doi:10.30773/PI.2017.08.17

5. Pereira T, Almeida PR, Cunha JPS, Aguiar A. Heart rate variability metrics for fine-grained stress level assessment. Comput Methods Programs Biomed. 2017;148: 71–80. doi:10.1016/J.CMPB.2017.06.018

6. RK R, R P, DP Z, B S, J EY, E D, et al. Accuracy of Wrist-Worn Activity Monitors During Common Daily Physical Activities and Types of Structured Exercise: Evaluation Study. JMIR Mhealth Uhealth. 2018;6. doi:10.2196/10338

7. Bent B, Goldstein BA, Kibbe WA, Dunn JP. Investigating sources of inaccuracy in wearable optical heart rate sensors. npj Digital Medicine 2020 3:1. 2020;3: 1–9. doi:10.1038/s41746-020-0226-6

8. Accuracy | Garmin. [cited 22 Aug 2021]. Available: https://www.garmin.com/en-US/legal/atdisclaimer/

9. Blevins CA, Weathers FW, Davis MT, Witte TK, Domino JL. The Posttraumatic Stress Disorder Checklist for DSM-5 (PCL-5): Development and Initial Psychometric Evaluation. J Trauma Stress. 2015;28: 489–498. doi:10.1002/JTS.22059

10. Löwe B, Decker O, Müller S, Brähler E, Schellberg D, Herzog W, et al. Validation and standardization of the generalized anxiety disorder screener (GAD-7) in the general population. Med Care. 2008;46: 266–274. doi:10.1097/MLR.0B013E318160D093

11. Mofaz M, Yechezkel M, Einat H, Kronfeld-Schor N, Yamin D, Shmueli E. Real-time sensing of war’s effects on wellbeing with smartphones and smartwatches. Communications Medicine 2023 3:1. 2023;3: 1–11. doi:10.1038/s43856-023-00284-y

12. Mofaz M, Yechezkel M, Guan G, Brandeau ML, Patalon T, Gazit S, et al. Self-Reported and Physiologic Reactions to Third BNT162b2 mRNA COVID-19 (Booster) Vaccine Dose. Emerg Infect Dis. 2022;28: 1375. doi:10.3201/EID2807.212330

13. Gepner Y, Mofaz M, Oved S, Yechezkel M, Constantini K, Goldstein N, et al. Utilizing wearable sensors for continuous and highly-sensitive monitoring of reactions to the BNT162b2 mRNA COVID-19 vaccine. Communications Medicine 2022 2:1. 2022;2: 1–8. doi:10.1038/s43856-022-00090-y
