# Supplementary material for: Social media impact and smartwatch monitoring: Prevalence and early markers of PTSD and anxiety following mass traumatic events
Source: PLOS Ment Health. 2025 Sep 10;2(9):e0000195. doi: 10.1371/journal.pmen.0000195 (PMC12798574; doi:10.1371/journal.pmen.0000195)
Supplement: S2 Appendix — (DOCX) [file pmen.0000195.s002.docx]

S2 Appendix for:

**Social Media Impact and Smartwatch Monitoring: Prevalence and Early Markers of PTSD and Anxiety Following Mass Traumatic Events**

Dan Yamin^1,2,3,†^, Shahar Lev-Ari^4,†^, Merav Mofaz^1^, Ron Elias^1^, Sharon Toker^5^, David Spiegel^6^, Matan Yechezkel^1^, Margaret L. Brandeau^2^, Erez Shmueli^1,3,7,†,*^

^1^ Department of Industrial Engineering, Tel Aviv University, Tel Aviv, Israel.

^2^ Department of Management Science and Engineering, Stanford University, Stanford, California, United States of America.

^3^ Wizermed D.H. LTD, Zoran, Israel.

^4^ Department of Health Promotion, Tel Aviv University, Tel Aviv, Israel.

^5^ Coller School of Management, Tel Aviv University, Tel Aviv, Israel.

^6^ Department of Psychiatry and Behavioral Sciences, Stanford University, Stanford, California, United States of America.

^7^ MIT Media Lab, MIT, Cambridge, Massachusetts, United States of America.

^†^ Contributed equally.

^*^ [shmueli@tau.ac.il](mailto:shmueli@tau.ac.il)

# **The online survey**

Participants will be asked to fill in an online survey that includes well-established PTSD surveys. The online survey incorporates The Post-traumatic Stress Disorder Checklist (PCL-5) is a self-report questionnaire designed to assess the 20 symptoms outlined in the DSM-5 criteria for PTSD. The PCL-5 serves various purposes, including screening individuals for PTSD and making provisional diagnoses [1]. It is widely used for clinical and research purposes; psychometrically, the PCL-5 demonstrates strong internal consistency (α = .94), high test-retest reliability (r = .82), and strong convergent validity (rs = .74 to .85) [1]. We will also utilize the General Anxiety Disorder (GAD) 7 item questionnaire (GAD-7) to identify cases of GAD along with measuring anxiety symptom severity. The tool is also widely used as a screening measure of panic, social anxiety, and PTSD. The questionnaire is considered valid, sensitive, and specific for the diagnosis of GAD in the general population [2]. Will use the Israeli Ministry of Health’s (IMOH) Hebrew translation of these surveys.

The online survey also includes questions regarding demographic information and consumption of news information.

PTSD diagnosis requires an interview conducted by a trained clinician. In this study, the outcome was derived from a screening tool. We defined PTSD as a total score of $\geq$ 33 on the PCL-5 questionnaire and meeting the DSM-5 diagnostic rule which requires at least: 1 B item (questions 1-5), 1 C item (questions 6-7), 2 D items (questions 8-14), 2 E items (questions 15-20).

**The English version of the online survey appears below:**

Dear participant,

The aim of the following questionnaire is to better understand the effects of the events that began on October 7.

The data will be used for statistical analysis as part of an academic study carried out by researchers from the Faculty of Medicine and the Faculty of Engineering from Tel Aviv University.

Your answers will help us learn about the effect of the "Swords of Iron" operation on the mental state of the citizens of the State of Israel and develop ways of coping with the effects of the war.

There are no correct or incorrect answers, but it is crucial to answer all questions truthfully.

The purpose of this questionnaire is not to diagnose any medical condition. If you are feeling unwell, we advise you to contact your primary care physician for further treatment.

Thank you for your valuable time and cooperation with the research team.

| **Table S2.1**. PTSD online survey. | |
| --- | --- |
| No. | Question |
| 1 | What is your age? |
| 2 | Please select all the statements that apply to you from the following list (you can select more than one statement)   - In the events, either my family members or I were injured. - One of my family members was abducted. - Either my family members or I were evacuated from our place of residence. - My source of income was affected by the events. - I am acquainted with people whom their family members or friends were killed, injured, or abducted during the events. - I am acquainted with people who were killed, injured, or abducted during the events. - I am acquainted with people who were evacuated from the place of residence (either from southern or northern Israel). - I am acquainted with people whom their source of income or their work capacity have been affected by the events. - None of the above. |
| 3 | During the first week of the war that began on October 7, how frequently did you consume news information (TV, news sites, social media) about the fighting events?   - I did not consume. - Less than an hour a day - 1-2 hours a day - 2-4 hours a day - 4-8 hours a day - More than 8 hours a day |
| 4 | During the last two weeks, how frequently did you consume news information (TV, news sites, social media) about the fighting events?   - I did not consume. - Less than an hour a day - 1-2 hours a day - 2-4 hours a day - 4-8 hours a day - More than 8 hours a day |
| 5 | Which platforms do you use to consume news information? (You can select more than one answer)   - TV - Facebook - Instagram - Twitter - TikTok - Telegram - WhatsApp - Newspapers - Word of mouth - Other, please specify |
| 6 | To the best of your recollection, how much were you exposed to gory videos depicting war events? (such as videos documenting the killings carried out on October 7, or the abductions of the captives)   - I was not exposed. - Occasionally (up to five times) - Many times (over five cases) |
| 7 | If you encountered gory videos of war events (such as videos documenting the killings carried out on October 7, or the abductions of the captives), on which digital platforms were you primarily exposed?   - TV - Facebook - Instagram - Twitter - TikTok - Telegram - WhatsApp - Other, please specify |
| 8 | Below is a list of problems that people sometimes have in response to a very stressful experience. Please read each problem carefully and then indicate how much you have been bothered by that problem in the past month following the events that began on October 7 (hereinafter the war events).   \| 1. In the past month, how much were you bothered by: \| Not at all \| A little bit \| Moderately \| Quite a bit \| Extremely \| \| --- \| --- \| --- \| --- \| --- \| --- \| \| 1. Repeated, disturbing, and unwanted memories of the war events? \|  \|  \|  \|  \|  \| \| 1. Repeated, disturbing dreams of the war events? \|  \|  \|  \|  \|  \| \| Suddenly feeling or acting as if the stressful experience of October 7/ war events, were actually happening again (as if you were actually back there reliving it)? \|  \|  \|  \|  \|  \| \| 1. Feeling very upset when something reminded you of the war events? \|  \|  \|  \|  \|  \| \| 1. Having strong physical reactions when something reminded you of war events (for example, heart pounding, trouble breathing, sweating)? \|  \|  \|  \|  \|  \| \| 1. Avoiding memories, thoughts, or feelings related to the war events? (referring to factors originating from within yourself and not external influences) \|  \|  \|  \|  \|  \| \| 1. Avoiding external reminders of the war events (for example, people, places, conversations, activities, objects, or situations)? \|  \|  \|  \|  \|  \| \| 1. Trouble remembering important parts of the war events? \|  \|  \|  \|  \|  \| \| 1. Having strong negative beliefs about yourself, other people, or the world (for example, having thoughts such as: I am bad, there is something seriously wrong with me, no one can be trusted, the world is completely dangerous)? \|  \|  \|  \|  \|  \| \| 1. Blaming yourself or someone else for the stressful experience or what happened after it? \|  \|  \|  \|  \|  \| \| 1. Having strong negative feelings such as fear, horror, anger, guilt, or shame? \|  \|  \|  \|  \|  \| \| 1. Loss of interest in activities that you used to enjoy? \|  \|  \|  \|  \|  \| \| 1. Feeling distant or cut off from other people? \|  \|  \|  \|  \|  \| \| 1. Trouble experiencing positive feelings (for example, being unable to feel happiness or have loving feelings for people close to you)? \|  \|  \|  \|  \|  \| \| 1. Irritable behavior, angry outbursts, or acting aggressively? \|  \|  \|  \|  \|  \| \| 1. Taking too many risks or doing things that could cause you harm? \|  \|  \|  \|  \|  \| \| 1. Being “superalert” or watchful or on guard? \|  \|  \|  \|  \|  \| \| 1. Feeling jumpy or easily startled? \|  \|  \|  \|  \|  \| \| 1. Having difficulty concentrating? \|  \|  \|  \|  \|  \| \| 1. Trouble falling or staying asleep? \|  \|  \|  \|  \|  \| |
| 9 | Over the last two weeks, how often have you been bothered by the following problems?   \|  \| Not at all \| Several days \| More than half the days \| Nearly every day \| \| --- \| --- \| --- \| --- \| --- \| \| 1. Feeling nervous, anxious, or on edge \|  \|  \|  \|  \| \| 1. Not being able to stop or control worrying \|  \|  \|  \|  \| \| 1. Worrying too much about different things \|  \|  \|  \|  \| \| 1. Trouble relaxing \|  \|  \|  \|  \| \| 1. Being so restless that it is hard to sit still \|  \|  \|  \|  \| \| 1. Becoming easily annoyed or irritable \|  \|  \|  \|  \| \| 1. Feeling afraid, as if something awful might happen \|  \|  \|  \|  \| |
| 10 | How many close friends do you have with whom you can share your feelings? (enter a numerical value only) |
| 11 | What is your sex?   - Male - Female - Other, please specify |
| 12 | What is your educational background (the last school you attended)?   - Elementary education - High School Diploma - Technical/Vocational Training - Bachelor's Degree - Master's Degree or higher |
| 13 | What is your marital status?   - Single - Married - Divorced or separated - Widow/er |
| 14 | How many individuals reside in your household, including yourself? |
| 15 | Do you have children? If yes, how many?   - Yes, how many: - No |
| 16 | Is either yourself or any of your immediate family members currently serving in the Israeli security forces? (For instance, in active duty or reserve service; you can select multiple options.)   - Myself - Child - Spouse - Parent - Sibling - Other, please specify - No |
| 17 | What is your religion?   - Jewish - Moslem - Christian - Druze - Other, please specify |
| 18 | If you identify with Judaism, how would you characterize your religious affiliation (i.e., religious level)?   - Secular - Conservative - National Religious - Ultra-Orthodox - Other (please specify) |
| 19 | As per the Central Bureau of Statistics, the average monthly income for a dual-income family in Israel is approximately NIS 20,000 gross (NIS 11,000 for a single breadwinner). How would you assess your income?   - Way below average - Slightly below average - Average - Slightly above average - Way above average |
| 20 | Where is your permanent place of residence? |
| 21 | Have you been diagnosed in the past (before the events of October 7) as suffering from one or more of the following syndromes:   - Post-traumatic stress disorder (PTSD) - Anxiety - Depression - I have not - I choose not to disclose |
| 22 | Did you experience attacks or symptoms of the syndrome in the three months preceding October 7?   - Yes - No - I choose not to disclose |

# **References**

1. Blevins CA, Weathers FW, Davis MT, Witte TK, Domino JL. The Posttraumatic Stress Disorder Checklist for DSM-5 (PCL-5): Development and Initial Psychometric Evaluation. J Trauma Stress. 2015;28: 489–498. doi:10.1002/JTS.22059

2. Löwe B, Decker O, Müller S, Brähler E, Schellberg D, Herzog W, et al. Validation and standardization of the generalized anxiety disorder screener (GAD-7) in the general population. Med Care. 2008;46: 266–274. doi:10.1097/MLR.0B013E318160D093
