# Supplementary material for: Social media impact and smartwatch monitoring: Prevalence and early markers of PTSD and anxiety following mass traumatic events
Source: PLOS Ment Health. 2025 Sep 10;2(9):e0000195. doi: 10.1371/journal.pmen.0000195 (PMC12798574; doi:10.1371/journal.pmen.0000195)
Supplement: S3 Appendix — (DOCX) [file pmen.0000195.s003.docx]

S3 Appendix for:

**Social Media Impact and Smartwatch Monitoring: Prevalence and Early Markers of PTSD and Anxiety Following Mass Traumatic Events**

Dan Yamin^1,2,3,†^, Shahar Lev-Ari^4,†^, Merav Mofaz^1^, Ron Elias^1^, Sharon Toker^5^, David Spiegel^6^, Matan Yechezkel^1^, Margaret L. Brandeau^2^, Erez Shmueli^1,3,7,†,*^

^1^ Department of Industrial Engineering, Tel Aviv University, Tel Aviv, Israel.

^2^ Department of Management Science and Engineering, Stanford University, Stanford, California, United States of America.

^3^ Wizermed D.H. LTD, Zoran, Israel.

^4^ Department of Health Promotion, Tel Aviv University, Tel Aviv, Israel.

^5^ Coller School of Management, Tel Aviv University, Tel Aviv, Israel.

^6^ Department of Psychiatry and Behavioral Sciences, Stanford University, Stanford, California, United States of America.

^7^ MIT Media Lab, MIT, Cambridge, Massachusetts, United States of America.

^†^ Contributed equally.

^*^ [shmueli@tau.ac.il](mailto:shmueli@tau.ac.il)

# **Statistical Analysis**

**Inferential Statistics in the Panel Study**- To examine the associations between PTSD (as assessed in the first PTSD survey), duration of news consumption during the first week following October 7, and the extent of exposure to gory videos, while controlling for other explanatory variables (age, sex, educational background, religious level, socioeconomic level, and PTSD background), we applied a logistic regression model:

| **(1)** | $\boldsymbol{ln}\left( \frac{\boldsymbol{p}}{\boldsymbol{1-p}} \right)\boldsymbol{\sim age + sex + educational background + religious level + socioeconomic level + PTSD background + duration of news consumption + gory video exposure,}$ |
| --- | --- |

where $\boldsymbol{p}$ represents the probability of probable PTSD. Age is treated as a continuous variable, while educational background, religious level, socioeconomic level, duration of news consumption, and gory video exposure are considered ordinal variables. Sex and PTSD background are categorized as Boolean variables.

Likewise, to examine the associations between moderate to severe anxiety (as assessed in the first PTSD survey), duration of news consumption during the two weeks before filling in the online PTSD survey, and the extent of exposure to gory videos, while controlling for other explanatory variables (age, sex, educational background, religious level, socioeconomic level, and anxiety background), we applied a logistic regression model:

| **(2)** | $\boldsymbol{ln} \left( \frac{\boldsymbol{p}}{\mathbf{1-}\boldsymbol{p}} \right)\boldsymbol{\sim}\boldsymbol{age}\mathbf{+}\boldsymbol{sex}\mathbf{+}\boldsymbol{educational} \boldsymbol{background}\mathbf{+}\boldsymbol{religious} \boldsymbol{level}\mathbf{+}\boldsymbol{socioeconomic} \boldsymbol{level}\mathbf{+}\boldsymbol{anxiety} \boldsymbol{background}\mathbf{+}\boldsymbol{duration} \boldsymbol{of} \boldsymbol{news} \boldsymbol{consumption}\mathbf{+}\boldsymbol{gory} \boldsymbol{video} \boldsymbol{exposure}\mathbf{,}$ |
| --- | --- |

where $\boldsymbol{p}$ represents the probability of moderate to severe anxiety. Age is treated as a continuous variable, while educational background, religious level, socioeconomic level, duration of news consumption, and gory video exposure are considered ordinal variables. Sex and anxiety background are categorized as Boolean variables.

For both models, we derived the odds ratios and their corresponding 95% confidence intervals from the values of the regression coefficients.

**Inferential Statistics in the Prospective Study**- To investigate the statistical significance of differences between the two periods and between the exhibition of probable PTSD and no exhibition, we used a mixed ANOVA design. For each of the above-noted 10 indicators, we used a separate mixed ANOVA test. In each test, the considered indicator served as the dependent variable. For the independent variables (main factors), the within-subjects factor was the period (the week prior to October 7, 2023 and the week after October 7, 2023). The between-subjects factor was the exhibition of probable PTSD (as assessed in the first PTSD survey).

More formally, for each of the 10 well-being indicators, the Mixed ANOVA model includes the two main factors and their interaction:

| **(3)** | $\boldsymbol{Indicator}\boldsymbol{\sim}\boldsymbol{period}\mathbf{+}\boldsymbol{probable} \boldsymbol{PTSD}\mathbf{+}\boldsymbol{period}\mathbf{*}\boldsymbol{probable} \boldsymbol{PTSD}\mathbf{,}$ |
| --- | --- |

To reduce the risk of inflated Type I errors due to multiple comparisons, we applied the Benjamini-Hochberg procedure.
