# Supplementary material for: Social media impact and smartwatch monitoring: Prevalence and early markers of PTSD and anxiety following mass traumatic events
Source: PLOS Ment Health. 2025 Sep 10;2(9):e0000195. doi: 10.1371/journal.pmen.0000195 (PMC12798574; doi:10.1371/journal.pmen.0000195)
Supplement: S4 Appendix — (DOCX) [file pmen.0000195.s004.docx]

S4 Appendix for:

**Social Media Impact and Smartwatch Monitoring: Prevalence and Early Markers of PTSD and Anxiety Following Mass Traumatic Events**

Dan Yamin^1,2,3,†^, Shahar Lev-Ari^4,†^, Merav Mofaz^1^, Ron Elias^1^, Sharon Toker^5^, David Spiegel^6^, Matan Yechezkel^1^, Margaret L. Brandeau^2^, Erez Shmueli^1,3,7,†,*^

^1^ Department of Industrial Engineering, Tel Aviv University, Tel Aviv, Israel.

^2^ Department of Management Science and Engineering, Stanford University, Stanford, California, United States of America.

^3^ Wizermed D.H. LTD, Zoran, Israel.

^4^ Department of Health Promotion, Tel Aviv University, Tel Aviv, Israel.

^5^ Coller School of Management, Tel Aviv University, Tel Aviv, Israel.

^6^ Department of Psychiatry and Behavioral Sciences, Stanford University, Stanford, California, United States of America.

^7^ MIT Media Lab, MIT, Cambridge, Massachusetts, United States of America.

^†^ Contributed equally.

^*^ [shmueli@tau.ac.il](mailto:shmueli@tau.ac.il)

**Additional results**

**Levels of PTSD in the panel study using the least conservative case definition**- In a secondary analysis, we considered a less conservative definition of PTSD, whereby it is identified by either a total score of 31 on the PCL-5 questionnaire or by meeting the DSM-5 diagnostic rule. According to this definition, the estimated prevalence of PTSD was 35.96% [95% CI: 34.05%-37.93%] in the survey conducted 7-8 weeks after October 7 and 24.73% [95% CI: 22.64%-26.89%] in the survey conducted seven months after October 7.

**Levels of PTSD and Anxiety in the prospective study**- We evaluated the extent to which the atrocities had post-traumatic effects on those indirectly exposed. To do so, we conducted a Post-traumatic Stress Disorder Checklist (PCL-5) survey among the 746 participants who were indirectly exposed to the atrocities and filled-in the PTSD survey from November 23, 2023, to December 3, 2023. We found that the estimated prevalence of PTSD was 16.22% (95% CI 13.67%-18.90%) using the most conservative case definition, and up to 29.49% (95% CI 26.27%-32.84%) with the least conservative definition. Similarly, using the General Anxiety Disorder (GAD) 7-item questionnaire among those not directly exposed, the estimated prevalence of anxiety among participants was 48.66% (95% CI 45.04%-52.28%), with 21.85% (95% CI 18.90%-24.80%) being moderate to severe, and is also lower to the level found in the panel study (as elaborated below).

| **Table A.** Cohorts’ characteristics. | | | |  |
| --- | --- | --- | --- | --- |
|  | **Prospective Cohort** | | **Panel study cohort** | |
|  | **Active participants pre-war period** | **Active participants after October 7, 2023** | **First online survey (6-7 weeks after October 7,2023)** | **Second online survey (7 months after October 7,2023)** |
| **Total** (%) | 4,797  (100%) | 1,751  (100%) | 2,536  (100%) | 1,773  (100%) |
| **Sex** |  |  |  |  |
| Female (%) | 2,387  (49.8%) | 898  (51.3%) | 1,340  (52.86%) | 933  (52.62%) |
| Male (%) | 2410  (50.2%) | 853  (48.7%) | 1195  (47.14%) | 840  (47.37%) |
| **Age** (years) |  |  |  |  |
| Mean | 45.6 | 48.6 | 39.86 | 42.52 |
| Std | 14.9 | 14.1 | 14 | 13.92 |
| Range | 20-90 | 20-88 | 18 - 77 | 18 - 75.1 |
| Median | 44 | 48 | 38 | 41 |
| **Age group** |  |  |  |  |
| 18-30 (%) | 944  (19.7%) | 198  (11.3%) | 820  (32.35%) | 425  (23.97%) |
| 31-50 (%) | 2,023  (42.2%) | 764  (43.6%) | 1049  (41.39%) | 790  (44.55%) |
| 51-64 (%) | 1,206  (25.1%) | 514  (29.4%) | 515  (20.32%) | 420  (23.68%) |
| $\geq$ 65 (%) | 624  (13.0%) | 275  (15.7%) | 150  (5.91%) | 138  (7.78%) |
| **Household income** |  |  |  |  |
| Below average (%) | 2,107  (43.9%) | 697  (39.8%) | 1,111  (43.8%) | 665  (37.5 %) |
| Average (%) | 883  (18.4%) | 374  (21.4%) | 751  (29.61%) | 483  (27.24%) |
| Above average (%) | 1,708  (35.6%) | 661  (37.7%) | 674  (26.57%) | 467  (26.33%) |
| Not reported (Skipped) | 99  (2.1%) | 19  (1.1%) | (-) | 158  (8.91%) |
| **Educational background** |  |  |  |  |
| Elementary education | 8  (0.01%) | 2  (0.01%) | 30  (1.18%) | 11  (0.6%) |
| High school diploma | 774  (16.1%) | 174  (9.9%) | 626  (24.68%) | 395  (22.27%) |
| Technical/Vocational Training | 1,061  (22.1%) | 380  (21.7%) | 670  (26.41%) | 481  (27.12%) |
| Academic degree | 2,937  (61.2%) | 1,194  (68.2%) | 1,210  (47.7%) | 886  (49.96%) |
| Not reported (Skipped) | 17  (0.03%) | 1  (0.0%) | (-) | (-) |
| **Extent of exposure^*^** |  |  |  |  |
| Direct | 14  (0.02%) | 14  (0.7%) | 52  (2.05%) | 71  (4%) |
| Affected | 42  (0.08%) | 43  (2.5%) | 143  (5.63%) | 125  (7.05%) |
| Indirect | 743  (15.5%) | 746  (42.6%) | 2,341  (92.31%) | 1,577  (88.94%) |
| Not reported (Skipped) | 3998  (83.3%) | 948  (54.1%) | (-) | (-) |
| **PTSD background** |  |  |  |  |
| Yes | 31  (0.06%) | 31  (1.8%) | 90  (3.54%) | 64  (3.6%) |
| Not | 738  (15.4%) | 741  (42.3%) | 2,298  (90.61%) | 1,603  (90.41 %) |
| Not reported | 4028  (84.0%) | 979  (55.9%) | 148  (5.83%) | 106  (5.97 %) |
| **Anxiety background** |  |  |  |  |
| Yes | 52  (1.1%) | 53  (3.0%) | 242  (9.54%) | 158  (8.91%) |
| Not | 717  (14.9%) | 719  (41.1%) | 2,146  (84.62%) | 1509  (85.11%) |
| Not reported (Skipped) | 4028  (84.0%) | 979  (55.9%) | 148  (5.83%) | 106  (5.97 %) |
| * The extent of exposure is determined by PTSD survey question 2 (see S2 Appendix). ‘Indirect’ encompasses all participants who, along with their immediate family, were not injured, killed, or abducted. 'Affected' refers to individuals who responded that their source of income was affected or were evacuated from their place of residence. ‘Direct’ includes individuals who responded that they or their immediate family were injured, killed, or abducted. | | | | |

| **Table B.** Logistic Regression model coefficients for PTSD within the participants of the panel study | | | |
| --- | --- | --- | --- |
| **Factor** | **Coefficient (95% CI)** | **P-value** | **Odds Ratio (95% CI)** |
| Age | -0.0198 (-0.028 to -0.012) | <0.001 | 0.98 (0.973 to 0.988) |
| Sex | 0.6678 (0.458 to 0.877) | <0.001 | 1.95 (1.581 to 2.405) |
| Educational background | 0.0774 (-0.026 to 0.181) | 0.141 | 1.081 (0.975 to 1.198) |
| Religious level | -0.1332 (-0.247 to -0.019) | 0.022 | 0.875 (0.781 to 0.981) |
| Socioeconomic level | -0.0876 (-0.175 to 0) | 0.051 | 0.916 (0.839 to 1) |
| PTSD background | 1.1679 (0.674 to 1.662) | <0.001 | 3.215 (1.962 to 5.267) |
| Duration of news consumption during the first week following October 7 | 0.0992 (0.022 to 0.177) | 0.012 | 1.104 (1.022 to 1.193) |
| Extent of exposure to gory videos | 0.2304 (0.077 to 0.384) | 0.003 | 1.259 (1.08 to 1.468) |

| **Table C.** Logistic Regression model coefficients for anxiety within the participants of the panel study | | | |
| --- | --- | --- | --- |
| **Factor** | **Coefficient (95% CI)** | **P-value** | **Odds Ratio (95% CI)** |
| Age | -0.0082 (-0.016 to -0.001) | 0.034 | 0.992 (0.984 to 0.999) |
| Sex | 0.8534 (0.653 to 1.054) | <0.001 | 2.347 (1.921 to 2.868) |
| Educational background | 0.0055 (-0.093 to 0.104) | 0.912 | 1.006 (0.911 to 1.11) |
| Religious level | -0.115 (-0.224 to -0.006) | 0.039 | 0.891 (0.799 to 0.994) |
| Socioeconomic level | -0.1091 (-0.193 to -0.025) | 0.011 | 0.897 (0.824 to 0.975) |
| Anxiety background | 0.9469 (0.647 to 1.247) | <0.001 | 2.578 (1.909 to 3.48) |
| Duration of news consumption during the two weeks before filling in the online PTSD survey | 0.2203 (0.145 to 0.296) | <0.001 | 1.246 (1.156 to 1.344) |
| Extent of exposure to gory videos | 0.2258 (0.083 to 0.369) | 0.002 | 1.253 (1.086 to 1.446) |

| **Table D.** Average difference between the baseline period and the week after October 7, 2023, for each well-being indicator among the participants of the prospective study | | | | |
| --- | --- | --- | --- | --- |
| **Well-being indicator** | **N** | **Baseline average value (95% CI)** | **Week after October 7, 2023, average value (95% CI)** | **Average difference** |
| Reported mood | 228 | 3.68 (3.59 to 3.77) | 2.81 (2.69 to 2.93) | -0.87 |
| Reported stress | 228 | 2.36 (2.26 to 2.46) | 3.28 (3.15 to 3.40) | 0.90 |
| Step counts | 621 | 7881.74 (7588.00 to 8175.47) | 6126.76 (5841.64 to 6411.89) | -1754.97 |
| Distance travelled in meters | 621 | 5983.14 (5675.98 to 6290.29) | 4605.25 (4325.89 to 4884.62) | -1377.89 |
| Reported sleep quality | 227 | 3.51 (3.41 to 3.60) | 3.14 (3.03 to 3.25) | -0.37 |
| Reported sleep time | 225 | 6.61 (6.49 to 6.74) | 6.38 (6.23 to 6.53) | -0.23 |
| Awake duration in seconds | 494 | 594.25 (544.84 to 643.66) | 687.35 (628.82 to 745.88) | 93.10 |
| Light sleep duration in seconds | 494 | 16031.23 (15767.75 to 16294.70) | 16312.04 (16008.71 to 16615.38) | 280.82 |
| Rem sleep in seconds | 494 | 4631.35 (4445.45 to 4817.24) | 4786.15 (4577.13 to 4995.16) | 154.80 |
| Deep sleep duration in seconds | 494 | 4313.71 (4116.09 to 4511.34) | 4304.18 (4085.71 to 4522.65) | -9.54 |

| **Table E.** Average difference between the baseline period and the week after October 7, 2023, for each well-being indicator among the participants of the prospective study, stratified by exhibition of PTSD. | | | | |
| --- | --- | --- | --- | --- |
| **Well-being indicator** | **PTSD*** | **Baseline average value** | **Week after October 7, 2023, average value** | **Average difference** |
| Reported mood | No | 3.71 (3.66 to 3.76) | 2.89 (2.83 to 2.96) | -0.82 |
|  | Yes | 3.55 (3.50 to 3.61) | 2.27 (2.20 to 2.34) | -1.28 |
| Reported stress | No | 2.29 (2.23 to 2.34) | 3.14 (3.07 to 3.21) | 0.85 |
|  | Yes | 2.67 (2.60 to 2.73) | 3.98 (3.92 to 4.04) | 1.31 |
| Step counts | No | 8021.87 (7844.06 to 8199.67) | 6402.28 (6223.12 to 6581.44) | -1619.59 |
|  | Yes | 7491.50 (7319.69 to 7663.31) | 5336.39 (5188.88 to 5483.90) | -2155.11 |
| Distance travelled in meters | No | 6258.60 (6056.52 to 6460.67) | 4925.72 (4733.44 to 5117.99) | -1332.88 |
|  | Yes | 5406.74 (5248.06 to 5565.42) | 3783.53 (3667.25 to 3899.81) | -1623.21 |
| Reported sleep quality | No | 3.58 (3.53 to 3.63) | 3.27 (3.21 to 3.32) | -0.31 |
|  | Yes | 3.37 (3.31 to 3.43) | 2.68 (2.62 to 2.74) | -0.70 |
| Reported sleep time | No | 6.66 (6.59 to 6.72) | 6.48 (6.41 to 6.55) | -0.17 |
|  | Yes | 6.35 (6.27 to 6.43) | 5.84 (5.72 to 5.96) | -0.51 |
| Awake duration in seconds | No | 594.21 (565.00 to 623.42) | 665.13 (629.72 to 700.53) | 70.92 |
|  | Yes | 574.45 (551.08 to 597.82) | 738.05 (709.66 to 766.45) | 163.60 |
| Light sleep duration in seconds | No | 15911.31 (15760.01 to 16062.60) | 16079.81 (15904.66 to 16254.95) | 168.50 |
|  | Yes | 16797.03 (16645.68 to 16948.39) | 17137.82 (16967.12 to 17308.52) | 340.79 |
| Rem sleep in seconds | No | 4730.07 (4612.17 to 4847.97) | 4781.87 (4655.31 to 4908.43) | 51.80 |
|  | Yes | 4559.48 (4461.32 to 4657.63) | 4661.21 (4549.23 to 4773.19) | 101.74 |
| Deep sleep duration in seconds | No | 4281.51 (4161.61 to 4401.40) | 4343.37 (4209.04 to 4477.69) | 61.86 |
|  | Yes | 4344.85 (4237.13 to 4452.57) | 4296.20 (4164.64 to 4427.75) | -48.65 |

| **Table F.** Mixed ANOVA results of each well-being indicator among the participants of the prospective study | | | |
| --- | --- | --- | --- |
| **Factor Name** | **Period** | **PTSD** | **Period * estimated PTSD prevalence sPTSD Interaction** |
| Reported mood | 0.000*** | 0.000*** | 0.010* |
| Reported stress | 0.000*** | 0.000*** | 0.010* |
| Step counts | 0.000*** | 0.031* | 0.200 |
| Distance travelled in meters | 0.000*** | 0.031* | 0.669 |
| Reported sleep quality | 0.000*** | 0.000*** | 0.010* |
| Reported sleep time | 0.036* | 0.003** | 0.180 |
| Awake duration in seconds | 0.017* | 0.488 | 0.287 |
| Light sleep duration in seconds | 0.339 | 0.031* | 0.734 |
| Rem sleep in seconds | 0.563 | 0.787 | 0.821 |
| Deep sleep duration in seconds | 0.563 | 0.890 | 0.734 |
| *** p<0.001, ** p<0.01, * p<0.05 | | | |

# **Additional results: continuous physiological measures**

To calculate the changes in smartwatch measures after October 7, 2023, for active participants in the prospective study, (Figs A and B), we performed the following steps. First, for each participant and each hour we calculated the difference between the mean value of the measure tested on that hour and that of the corresponding hour in a week prior (keeping the same day of the week and same hour during the day). If such data were not recorded (e.g., a participant did not wear the smartwatch in the same period before and after vaccination), we excluded the participant from this analysis. Then, we aggregated each hour's differences over all participants to calculate a mean difference and the associated 95% confidence interval.

| **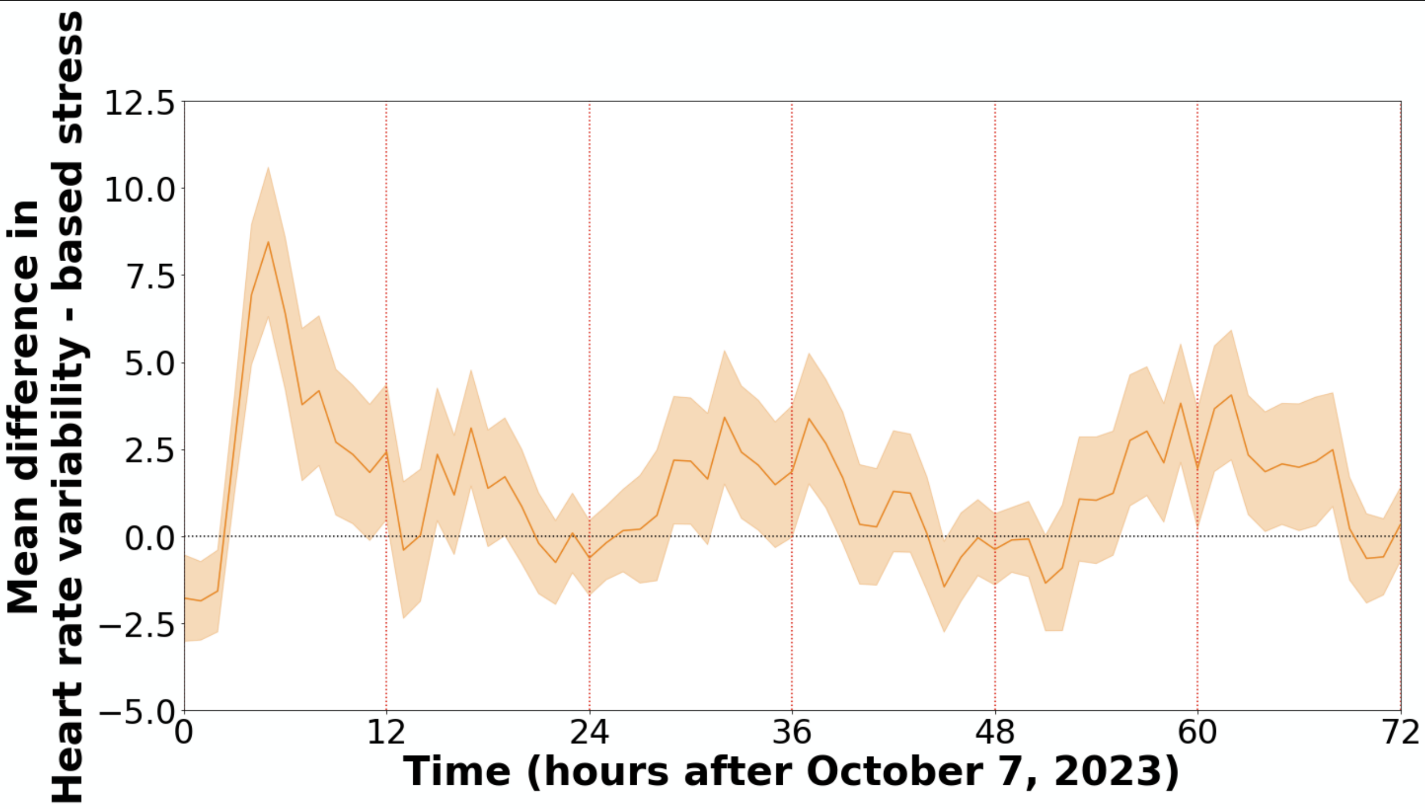** |
| --- |
| **Fig A**. HRV-based stress reaction to October 7, 2023, events as recorded by the smartwatches. The figures show the mean difference between the baseline in the week that preceded October 7^th^, and the week after period in terms heart rate variability-based stress (n=512). Mean values are depicted as solid lines, and 95% confidence intervals are presented as shaded regions. We present values starting from 12:00 AM of October 7 (the events started at 6:30). |

| **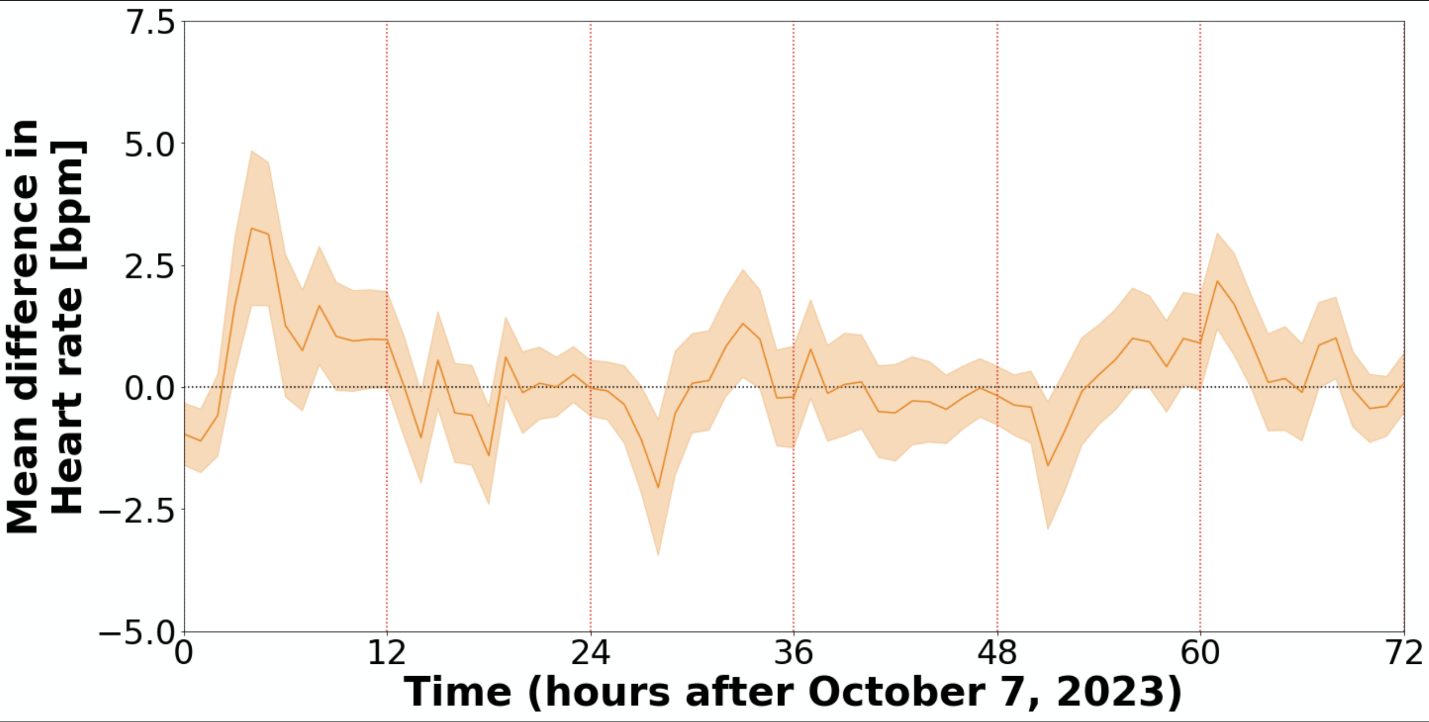** |
| --- |
| **Fig B**. Heart rate reaction to October 7, 2023, events as recorded by the smartwatches. The figures show the mean difference between the baseline in the week that preceded October 7^th^ and the week after period in terms heart rate (n=495). Mean values are depicted as solid lines, and 95% confidence intervals are presented as shaded regions. We present values starting from 12:00 AM of October 7 (the events started at 6:30). |
